# Double DAP-seq uncovered synergistic DNA binding of interacting bZIP transcription factors

**DOI:** 10.1038/s41467-023-38096-2

**Published:** 2023-05-05

**Authors:** Miaomiao Li, Tao Yao, Wanru Lin, Will E. Hinckley, Mary Galli, Wellington Muchero, Andrea Gallavotti, Jin-Gui Chen, Shao-shan Carol Huang

**Affiliations:** 1 Center for Genomics and Systems Biology, Department of Biology, New York University, New York, NY 10003 USA; 2 Biosciences Division, Oak Ridge National Laboratory, Oak Ridge, TN 37831 USA; 3 Waksman Institute of Microbiology, Rutgers University, Piscataway, NJ 08854-8020 USA

**Keywords:** Transcriptional regulatory elements, Plant molecular biology, Plant signalling

## Abstract

Many eukaryotic transcription factors (TF) form homodimer or heterodimer complexes to regulate gene expression. Dimerization of BASIC LEUCINE ZIPPER (bZIP) TFs are critical for their functions, but the molecular mechanism underlying the DNA binding and functional specificity of homo- *versus* heterodimers remains elusive. To address this gap, we present the double DNA Affinity Purification-sequencing (dDAP-seq) technique that maps heterodimer binding sites on endogenous genomic DNA. Using dDAP-seq we profile twenty pairs of C/S1 bZIP heterodimers and S1 homodimers in *Arabidopsis* and show that heterodimerization significantly expands the DNA binding preferences of these TFs. Analysis of dDAP-seq binding sites reveals the function of bZIP9 in abscisic acid response and the role of bZIP53 heterodimer-specific binding in seed maturation. The C/S1 heterodimers show distinct preferences for the ACGT elements recognized by plant bZIPs and motifs resembling the yeast GCN4 *cis*-elements. This study demonstrates the potential of dDAP-seq in deciphering the DNA binding specificities of interacting TFs that are key for combinatorial gene regulation.

## Abstract (web summary)

Here, the authors describe a new method to study how some proteins work together to control gene activity. They show that certain protein pairs can recognize new DNA sequences that they can’t recognize individually and control a wider range of genes.

## Introduction

Sequence-specific transcription factors (TF) regulate the expression of their target genes by recognizing short DNA sequences known as transcription factor binding sites (TFBS). The specificity of TF-DNA interactions can also be affected by genome and epigenome context or protein cofactors^1–6^. In particular, most TFs work within a complex or interact with other proteins to regulate gene expression in vivo^7^. Interactions of TFs with other TFs could alter, enhance or repress DNA binding activity depending on their cooperative, synergistic or competitive relationships^7–9^. Importantly, for many TFs across diverse structural families, TF interactions modify the recognition motifs of individual TFs, including alternate motif spacing and orientation^10^. Therefore, elucidating how the DNA binding specificity of a TF is altered by interaction with another TF is key to accurate understanding of combinatorial gene regulation and TF functions.

Homo- and heterodimerization are an important feature of DNA recognition and regulatory function for many TFs^1,11,12^. A well-known example in plants, the AUXIN RESPONSE FACTOR (ARF) family of TFs, requires dimerization to bind DNA efficiently. Structural analysis supports a model where ARF dimers interact with DNA in multiple configurations, and the DNA binding affinity of a dimer can be much higher than that of each of the two monomers^3^. In another case, the BASIC HELIX-LOOP-HELIX (bHLH) TFs bind DNA as homodimers, while heterodimerization between HLH and bHLH inhibits its DNA binding activity^13–15^. Therefore, heterodimerization not only increases the combinatorial complexity for a limited number of TFs, but also enhances their functional specificity^16,17^.

The BASIC LEUCINE ZIPPER (bZIP) proteins are dimerizing TFs found in all eukaryotes^16^. Dimerization of the DNA-binding domain occurs via the leucine zipper domain that positions the “basic” region of each monomer in contact with DNA^18^. The expansion of the bZIP family by genome duplication appears to have driven a strong tendency for homo- and heterodimerization between members, forming a bZIP dimer-mediated regulatory network^16,19^. The dimerization properties of this family play a significant role in regulating the overlapping and unique biological functions of the family members in both plants and animals^16,20^. For instance, the human TF AP-1 is formed by a mixture of homo- and heterodimers of JUN and FOS proteins where the dimer composition defines the pattern of target gene expression^21^. The human bZIP ATF3 acts as a repressor as a homodimer but becomes an activator when it heterodimerizes with JUN^22^. A systematic survey of DNA binding specificities of human bZIP heterodimers revealed that many heterodimers targeted new types of DNA binding motifs that were not bound by either of the interacting partners^11^. In plants, studies started in the 1990s explored the DNA binding activities of different bZIPs using a gel-shift approach which provided a foundation for subsequent investigations of DNA sequence recognition by plant bZIPs^23–26^. Among the key findings were that plant bZIPs preferred *cis-*elements containing the ACGT sequence, such as the G-box (CACGTG), C-box (CACGTC), and A-box (TACGTA)^23^, and nucleotides flanking these ACGT elements played important roles in determining specificity. In addition to the ACGT elements, a handful of other *cis*-acting elements, such as the ACT element (ACTCAT/ATGAGT), have also been identified as important binding sites for heterodimers formed by plant group C and S1 bZIPs^27–30^.

In the model plant *Arabidopsis thaliana*, 78 bZIP proteins are classified into thirteen phylogenetic groups^20^. Heterodimerization has been widely observed among the paralogous groups C and S1^31–34^. Genetic studies revealed that combinatorial regulation by these two groups is essential to maintain plant growth and development, as well as response to biotic and abiotic stresses, such as in low energy environments^33,35–37^. For example, bZIP53 (group S1) and bZIP10 (group C) interactions strongly enhance the expression of *PROLINE DEHYDROGENASE* (*ProDH)*^38^, which catalyzes the breakdown of proline as part of amino acid recycling to support energy demand^28,39^. This activation is achieved by increases in bZIP53 and bZIP10 heterodimer binding at an ACT element that resembles a preferred binding sequence of GCN4^27–30,40,41^, a well-characterized bZIP from unicellular yeast that diverged from *Arabidopsis* one billion years ago^42–44^. *ASPARAGINE SYNTHETASE1* (*ASN1*), which encodes a glutamine-dependent central enzyme in asparagine synthesis^45^, is also regulated by the C/S1 heterodimer via a G-box motif in its promoter region^33,35,38,45^. These findings suggest that bZIP C/S1 heterodimerization gives rise to important functions and regulatory mechanisms that are distinct from the homodimers.

Despite the importance of heterodimerization to the functions of the C/S1 bZIPs, the molecular mechanisms underlying the functional diversity and specificity of different homo- and heterodimers remains elusive. The pairs interact promiscuously in several assays^31–34^ and have a high degree of co-expression across tissue types and conditions^46^, so neither protein–protein interaction nor co-expression patterns are sufficient to explain the unique functions for the heterodimers. Here we extended our previously published method DAP-seq^47,48^, where an in vitro expressed TF is incubated with genomic DNA library to identify genome-wide TFBS for individual TFs, to allow mapping of binding sites on endogenous genomic DNA for interacting TFs. We generated binding site maps for twenty pairs of the C/S1 homo- and heterodimers and found that a substantial number of binding sites for the heterodimers were not shared with the homodimers, and that these unique binding events were associated with novel transcriptional responses and predicted biological processes. Importantly, the heterodimer- and homodimer-specific binding events could be distinguished by the presence of the classic ACGT elements *vs*. the GCN4-like elements. Compared to experiments that interrogated binding of interacting TFs on synthetic oligonucleotides that contained limited genomic context^10,11^, our binding site maps captured the sequence diversity and binding site context of real genomic DNA. Therefore, our dataset provides a baseline for determining heterodimer targets based on DNA binding specificities, and the rules that we uncovered for combinatorial TF binding contribute valuable insights for understanding combinatorial gene regulation.

## Results

### dDAP-seq identified in vitro, genomic-context binding sites of bZIP C/S1 heterodimers

**Fig. 1:**
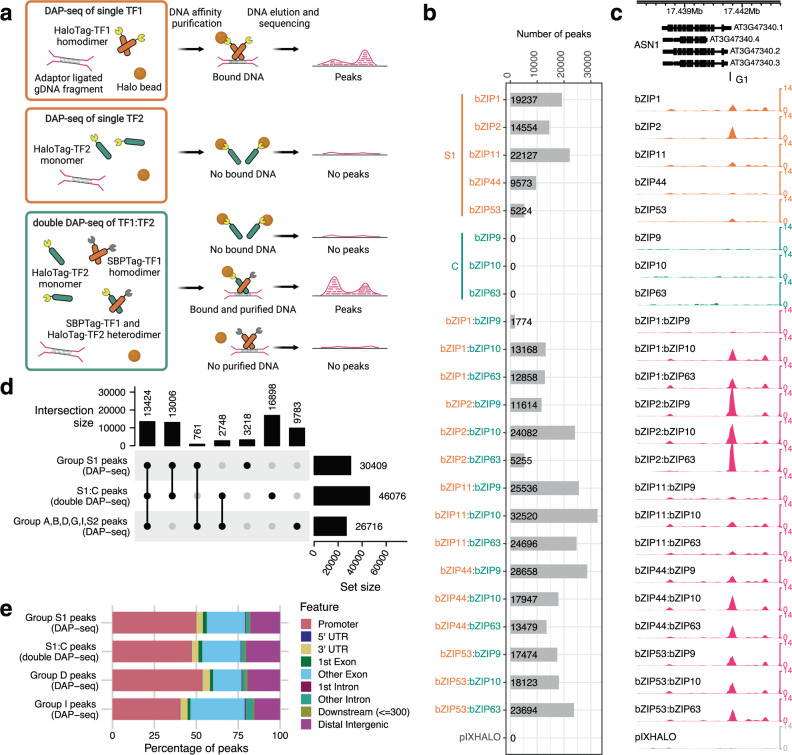
Systematic identification of bZIP C/S1 bZIP homodimer and heterodimer binding sites by DAP-seq and dDAP-seq. **a** Schematic of DAP- and dDAP-seq. Top panel: in DAP-seq, an in vitro expressed, HaloTag-fused TF (HaloTag-TF1) forms homodimers that bind to genomic DNA (gDNA) fragments ligated to Illumina-compatible sequencing adapters. The TF-DNA complex is purified by HaloTag ligand-coupled magnetic beads, from which the bound gDNA is eluted and sequenced. Mapping the sequencing reads to the reference genome allows identification of TF binding location as peak regions of significant read enrichment. Middle panel: Performing DAP-seq for a TF that cannot bind DNA by itself does not produce any peaks. Bottom panel: in double DAP-seq, two TFs fused to SBPTag and HaloTag separately, SBPTag-TF1 and HaloTag-TF2, are co-expressed in vitro and allowed to form heterodimers. HaloTag ligand-coupled magnetic beads are used to purify the complex of SBPTag-TF1:HaloTag-TF2 with the bound DNA. Although the beads can pull down the HaloTag-TF2 monomer, no peaks will be detected for them without the bound DNA. Created with BioRender.com. **b** Number of peaks from all pairs of C/S1 bZIPs detected by DAP-seq and dDAP-seq. S1 bZIPs (bZIP1, 2, 11, 44, and 53) are indicated by orange color, and C bZIPs (bZIP9, 10, and 63) are indicated by teal color. **c** DAP-seq and dDAP-seq binding profiles of C/S1 homodimers and heterodimers at the known target gene *ASN1* (AT3G47340). G1 marks a previously mapped functional G-box^36^. **d** Upset plot comparing the peak overlap between S1 homodimers, S1:C heterodimers and bZIPs from group A, B, D, G, I, and S2. The dot plot lists the possible combinations between the different bZIP groups. The vertical bar plot at the top reports the number of peaks bound by each combination, and the horizontal bar plot on the right shows the total number of peaks bound by each bZIP group. **e** Distribution of binding sites relative to genomic features for the bZIP homodimers and heterodimers. Promoter regions were defined as ±1 kb from the TSS.

Functional characterization of bZIP TFs is challenging due to the high degree of redundancy and synergistic interactions among family members^20,49–51^. We sought to deconvolute some of this complexity by studying the genome-wide binding profiles of the homodimers and heterodimers formed by members in the groups C and S1. We hypothesized that the functional specificities could be mediated by variation of DNA binding specificities between the pairs, which had not been investigated systematically due to technical limitations. Previously we published the DAP-seq method that used in vitro expressed TFs to interrogate DNA libraries constructed from naked genomic DNA^47,48^. Compared to methods that assay protein binding on synthetic oligonucleotides, such as systematic evolution of ligands by exponential enrichment (SELEX)^10^ and protein binding microarray (PBM)^52^, DAP-seq binding events occur in the context of endogenous genome sequence and DNA chemical modification. In contrast to in vivo methods such as chromatin immunoprecipitation followed by sequencing (ChIP-seq), which finds but does not distinguish direct and indirect binding events^53,54^, DAP-seq reports binding sites from direct interaction between the expressed TF and genomic DNA. We first applied DAP-seq to group S1 members (bZIP1, bZIP2, bZIP11, bZIP44, and bZIP53; Fig. 1a) and in agreement with their previously described capacity to bind DNA as homodimers^32,49^, we successfully identified 5200–22,000 peaks (regions of read enrichment with *q*-value ≤ 0.01) for each of the members (Fig. 1b). In contrast, no peaks were found for any of the four group C members (bZIP9, bZIP10, bZIP25, and bZIP63) when tested alone using the same DAP-seq strategy, despite the presence of a highly conserved bZIP DNA-binding domain (Fig. 1b) and previous reports of DNA binding^34,38,55,56^. To ensure our inability to detect peaks for group C members was not caused by poor protein expression or protein misfolding, we performed in vitro pull-down assays using similar conditions to DAP-seq and detected protein–protein interactions between C and S1 bZIPs that were consistent with most published results from cellular assays^31,32,57^ (Supplementary Fig. 1a–d). These data, together with reports that heterodimer formation between C and S1 bZIPs could increase DNA binding^32,33,38,57,58^, led us to hypothesize that performing DAP-seq for C bZIPs in the presence of S1 may allow detection of DNA binding sites and motivated us to develop a new DAP-seq technique for detecting DNA binding events of interacting TFs which we refer to as double DAP-seq (dDAP-seq). As illustrated in Fig. 1a, instead of expressing only one TF fused to a HaloTag as in the published DAP-seq protocol, in dDAP-seq we simultaneously expressed two TFs fused to different affinity tags, TF1 to a Streptavidin-Binding Peptide Tag (SBPTag-TF1) and the other TF2 to a HaloTag (HaloTag-TF2), with SBPTag-TF1 expressed at higher level to drive HaloTag-TF2 to heterodimerize with SBPTag-TF1. After incubating the expressed proteins with fragmented genomic DNA, we used HaloTag ligand-coupled magnetic beads to specifically capture proteins or protein complexes that contain Halo-Tag TF2 along with the bound DNA fragments, and sequenced the recovered DNA. Since most HaloTag-TF2 protein form are in heterodimers with SBPTag-TF1 and HaloTag-TF2 proteins on their own do not produce significant read enrichment, the resulting peaks revealed the locations of the heterodimer binding events throughout the whole genome. We note that dDAP-seq is particularly useful for testing heterodimerization when one or both of the TFs on their own do not bind DNA or do not produce peaks in DAP-seq. For example, to test for heterodimerization among bZIP C/S1 family members, HaloTag-TF2 corresponded to a group C bZIP, which do not produce peaks in single protein DAP-seq. SBPTag-TF1 corresponded to a group S1 bZIP, which could bind DNA as a homodimer to produce peaks in DAP-seq but was not captured in dDAP-seq in the HaloTag affinity purification step. As long as sufficient DNA is added for all the proteins in the binding reaction, comparing dDAP-seq binding profiles of C and S1 bZIP to DAP-seq of S1 bZIP would reveal heterodimer-specific DNA binding.

Using dDAP-seq, we comprehensively profiled the genome-wide binding events of twenty heterodimer pairs between five group S1 and four group C members with corresponding empty vector controls (Supplementary Data 1). Henceforth we use “C/S1” to refer to dimers among these nine bZIPs, including S1 homo- and S1 and C heterodimers, and “S1:C” to exclusively refer to the S1 and C heterodimers. In contrast to the DAP-seq experiments for C bZIPs alone that found no significant peak enrichment, dDAP-seq of the S1:C heterodimers revealed between 1700 and 33,000 peaks (Fig. 1b). In the few cases where the interaction was weak or undetected, such as the interactions between bZIP25 and four S1 bZIPs (bZIP1, bZIP2, bZIP11, and bZIP44) potentially resulting from truncated bZIP25 protein (Supplementary Fig. 1f–i), dDAP-seq detected no stable binding signal and therefore these pairs were excluded from downstream analysis. Figure 1c shows an example of peaks produced by DAP-seq and dDAP-seq in the promoter of *ASN1*, known to be activated by bZIP53 in transient protoplast activation assays, and the functional G-box motif previously mapped^35,36^. Data from dDAP-seq were highly reproducible, where Pearson correlations between replicates ranged from 0.74 to 0.98 (Supplementary Fig. 2a) and peaks identified from two replicates and threes replicates overlap by more than 95% (Supplementary Fig. 2b). Considering the contrasting reports of DNA binding by C homodimers, we further confirmed that dDAP-seq peaks were indeed S1:C heterodimer binding sites by performing sequential DNA affinity purification (sDAP-seq) using SBPTag- and HaloTag-fused bZIP53 and bZIP63. dDAP-seq peaks for bZIP53:bZIP63 showed strong central enrichment of reads from sDAP-seq of bZIP53:bZIP63, a pattern that was not observed for reads from sDAP-seq of bZIP63:bZIP63 (Supplementary Fig. 2c). Furthermore, the bZIP53:bZIP63 heterodimer-specific peaks, identified by differential binding analysis that will be detailed in later sections, also had increase binding signal comparing sDAP-seq of bZIP53:bZIP63 to bZIP53:bZIP53 (Supplementary Fig. 2d–f). These results support that dDAP-peaks were bound by bZIP53:bZIP63 heterodimers, but not by bZIP63 monomers or homodimers. The complete genome-wide binding site maps are provided at http://hlab.bio.nyu.edu/projects/bzip_code.

Overall, a substantial fraction of S1:C heterodimer binding sites were not shared with binding sites of homodimers of S1 and other bZIP groups (Fig. 1d). We compared our binding site data for fifteen S1:C heterodimer pairs (dDAP-seq) and five S1 homodimers (DAP-seq) to published, single TF DAP-seq data for 21 *Arabidopsis* bZIPs in groups A, B, D, G, I, and S2^47^. Combining all these genome-wide binding site maps resulted in a total of 59,838 binding events. Among these, 13,424 peaks (22.4%) were common between the S1:C heterodimers and homodimers of other bZIP groups including S1, reflecting the conserved DNA binding preferences of the bZIP family. Among the 46,076 peaks bound by the S1:C heterodimers, 26,430 peaks (57.4%) were shared with the S1 homodimers, which may represent overlapping DNA recognition sites and redundant functions between heterodimers and homodimers. Another 2748 (6.0%) S1:C heterodimer peaks overlapped with binding sites of other bZIP groups, while 16,898 (36.7%) were new peaks not shared with group S1 or any other bZIP groups. The formation of unique binding sites by S1:C heterodimers indicates potentially cooperative functions among C and S1 bZIPs with a different binding specificity compared to S1 alone. The binding locations of homo- and heterodimers relative to genomic features are similar to those of other bZIP groups, with about half of all binding events located within 2 kb centered at the transcription start site (TSS; Fig. 1e).

### Characteristics of genome-wide binding profiles of C/S1 bZIP dimers

**Fig. 2:**
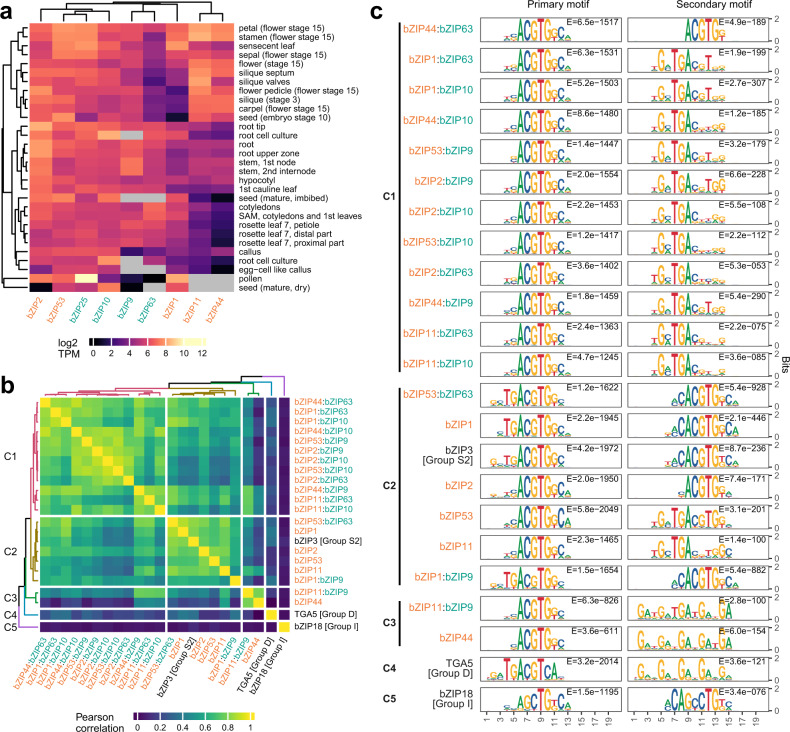
Overview of genome-wide binding correlation and DNA sequence motifs of C/S1 bZIP dimers. **a** S1 (labeled in orange color) and C (labeled in teal color) bZIPs are co-expressed in multiple *Arabidopsis* tissues at different developmental stages. **b** Hierarchical clustering using the Pearson correlation matrix of genome-wide binding profiles revealed three major clusters for C/S1 dimers (Cluster C1, C2, C3) that were separated from representative bZIPs from other groups, TGA5 (group D) in Cluster C4 and bZIP18 (group I) in Cluster C5. Pearson correlations were computed using log2-transformed normalized read counts on a consensus peak set of 8505 peaks where each peak is in the top 3000 most enriched peaks in each replicate (see “Methods” section). No statistical tests were used. **c** PWM models of the most enriched (primary) and the second most enriched (secondary) motifs discovered from the 1000 strongest bound peaks for each C/S1 bZIP homodimer and heterodimer. Cluster memberships correspond to those from **b**.

To determine the potential tissues and developmental stages where C/S1 dimerization may occur, we examined the co-expression patterns of S1 and C genes. We observed that each pair is co-expressed in multiple tissues at different developmental stages^59^ (Fig. 2a), indicating the different S1:C heterodimers could be formed in multiple tissues. This raises the possibility that the functions of different pairs of S1:C heterodimers are redundant and suggests that expression patterns alone provide limited information about functional differences between the pairs. Thus, we could not use transcript expression patterns alone to implicate a functional role for S1:C heterodimers in tissues.

Alternatively, we took a DNA-centric approach to narrow the context in which S1:C heterodimers may act. Since heterodimerization is known to alter DNA binding specificities for many bZIP proteins, we first investigated the relationships of DNA binding site locations among the C/S1 homodimer and heterodimers. To do this we calculated the Pearson correlation of genome-wide binding profiles between each pair of C/S1 dimers with representatives from other groups as outgroups and performed hierarchical clustering of the correlation matrix (Fig. 2b). We observed three major clusters for the C/S1 dimers that were separated from the two outgroup bZIPs, TGA5 (group D) and bZIP18 (group I). Cluster C1 contained twelve pairs of heterodimers, while the rest of the heterodimers and five homodimers were grouped together in Cluster C2 and Cluster C3. When we performed de novo motif discovery with the most enriched 1000 peaks bound by each homo- and heterodimer, we found the most significant motifs from all the experiments shared a core ACGTG binding sequence (Fig. 2c). Peak sequences for many of the S1:C heterodimers were also enriched for a secondary motif containing the sequence TGAC, which was absent in most of S1 homodimers (Fig. 2c). For example, 397 out of the top 1000 peaks (39.7%) for the heterodimer bZIP53:bZIP10 contained the TGAC motif. Remarkably, the patterns of occurrence of secondary motifs largely coincided with the clusters formed by genome-wide binding correlation (Fig. 2b). All the heterodimers in Cluster C1, except for bZIP44:bZIP63, had enriched TGAC secondary motifs. The homodimers and heterodimers in Cluster C2, except for bZIP11, had enriched ACGTG secondary motifs. Cluster C3, consisting of only the bZIP11:bZIP9 heterodimer and the bZIP44 homodimer, had G-rich secondary motifs. Therefore, both binding profile comparison and motif discovery results suggest that heterodimerization with group C changes the DNA binding preference of group S1 bZIPs, potentially mediating their specific regulatory function.

### dDAP-seq revealed direct binding at differentially expressed genes regulated by C/S1 dimers

**Fig. 3:**
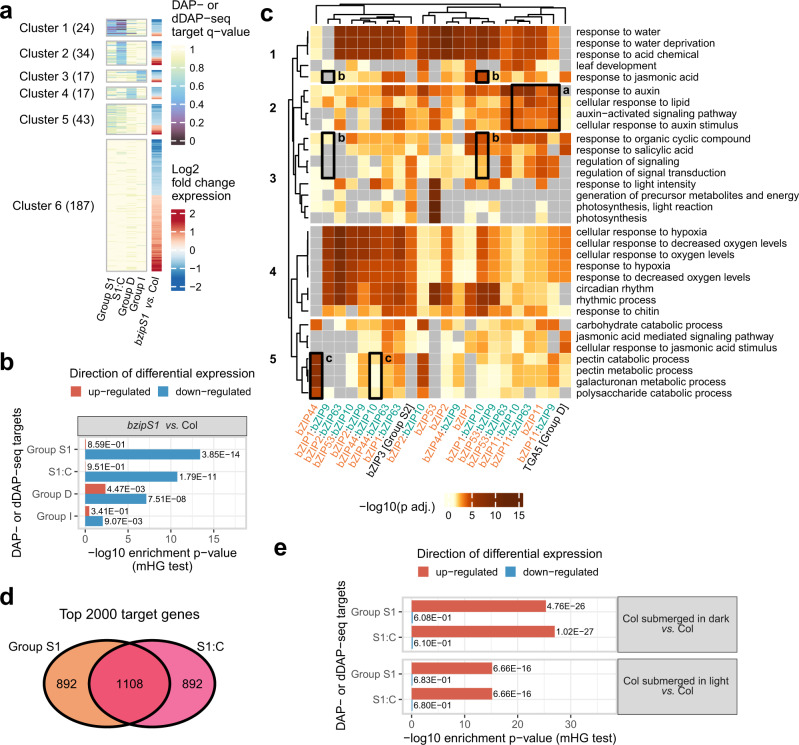
dDAP-seq identifies direct target genes of C/S1 bZIPs and suggest possible functions. **a** Distribution of DAP- and dDAP-seq target gene scores for the differentially expressed genes in *bzipS1* mutants. **b** Significance of association between DAP- and dDAP-seq target gene scores and DEGs in the *bzipS1* mutant. *P*-values shown were calculated by the minimal hypergeometric (mHG) test for gene set enrichment without multiple comparison correction. **c** Comparison of enriched GO terms for predicted target genes for S1 homodimers and S1:C heterodimers. Enriched GO terms for top 2000 target genes determined from DAP- and dDAP-seq peaks of C/S1 dimers and representatives from group D (TGA5) and group S2 (bZIP3). Gray color indicates no significant association was found for that GO term (adjusted *P* > 0.05). Labeled boxes correspond to discussion in text. *P*-values plotted were computed by hypergeometric tests (one-sided Fisher exact tests) and corrected for multiple comparison by the Benjamini and Hochberg method. **d** Venn diagram comparing the top 2000 target genes between the S1 homodimers and S1:C heterodimers. **e** Significance of association between DAP- and dDAP-seq targets with DEGs in response to submergence. *P*-values shown were calculated by the mHG test for gene set enrichment without multiple comparison correction.

To determine whether the S1:C heterodimer binding events identified by dDAP-seq could regulate gene expression, we compared the differentially expressed genes (DEGs) from RNA-seq of *bzipS1* quintuple mutant rosette leaves under starvation^37^ to three sets of target genes based on DAP- or dDAP-seq binding: the S1:C heterodimer targets from dDAP-seq, S1 homodimer targets from DAP-seq, and as a control the targets of group D and I bZIPs from DAP-seq. Since S1:C heterodimerization was induced by starvation^37,60^, we reasoned that DEGs in *bzips1* vs. wild type under starvation would include targets of S1:C heterodimers. For each set of DAP-seq or dDAP-seq binding peaks, we calculated the target scores for all the protein coding genes in the genome using the ClosestGene method in TFTargetCaller^61^. This method computed a score for each gene based on the overall distribution of distances between peaks to genes in a particular peak dataset and performed well in comparison to several commonly used peak-to-gene assignment approaches including window-based methods^61^. The 322 DEGs in the *bzipS1* mutant were grouped into six clusters based on the target score q-values (lower value is more significant; Fig. 3a Cluster 1–6; Supplementary Data 2). Cluster 1 contained 24 genes that were shared targets of S1 homodimers and S1:C heterodimers but were not enriched in targets of group D or I. Notably, 34 genes in Cluster 2 were uniquely enriched for S1:C heterodimer targets but not the targets of other groups. Cluster 3 and Cluster 4 genes displayed specific binding by group I and D, respectively. While Cluster 5 showed weak binding for both S1 and S1:C, Cluster 6 genes showed no enrichment from any of these bZIP groups, possibly representing indirect targets. We further noted that the genes in Cluster 1–5 were mostly down regulated in the *bzipS1* mutant (Fig. 3a), suggesting that binding by S1 bZIPs are correlated with gene activation. To quantify the significance of association between the DAP- and dDAP-seq binding target scores and gene expression changes in the *bzipS1* mutant, we performed a Gene Set Enrichment Analysis (GSEA)-style calculation for the target gene score and the DEGs, using the minimal hypergeometric (mHG) test that is more powerful than the standard one-sided Kolmogorov-Smirnov test in GSEA^62^. We found that the targets identified for S1 homodimers and S1:C heterodimers were the most enriched for the down-regulated DEGs, and the targets from group D and group I were much less significantly enriched (Fig. 3b). These results suggest that DAP- or dDAP-seq predicted targets of group C/S1 dimers are transcriptionally regulated by the S1 bZIPs in planta, identifying the DNA binding targets of homodimers and heterodimers for future functional studies.

We previously showed that single DAP-seq reported binding events were highly consistent with the in vivo direct binding sites identified by ChIP-seq and that target genes significantly overlapped with those obtained from TF overexpression experiments^47^. Since the S1 bZIPs can form homo- and heterodimers to bind DNA, it is unclear how the heterodimer binding found by dDAP-seq relate to the target genes found by ChIP-seq and overexpression of the S1 bZIPs. The targets for bZIP1 (group S1), a master regulator in nitrogen response, were previously investigated by ChIP-seq and an inducible overexpression assay in protoplasts known as TARGET (transient assay reporting genome-wide effects of transcription factors)^63^. A comparison of ChIP-seq and time-course TARGET data, which implicated transient binding events, showed that bZIP1 direct target genes fell into three classes: poised (Class I), stable (Class II), and transient (Class III). Each gene in the three classes was either activated (IA, IIA, or IIIA) or repressed (IB, IIB, or IIIB). Using these target genes, we plotted the normalized sequencing reads from dDAP-seq within the 2 kb region centered at the TSS (Supplementary Fig. 3). For both bZIP1 homo- and heterodimers, we observed strong read enrichment in regions immediately upstream of the TSS for many of the bZIP1 targets, suggesting binding at the proximal promoter could regulate the expression of these genes. More importantly, both DAP- and dDAP-seq showed strong binding at the promoters of transient targets of Class III, which are regulated by bZIP1 overexpression but could not be detected in ChIP-seq at a single time point^63^. Furthermore, we observed stronger binding signals by both bZIP1 homo- or heterodimers at bZIP1 activated targets than at the repressed targets. The highly similar homo- and heterodimer binding profiles at the bZIP1-regulated targets suggest that those targets may also be regulated by S1:C heterodimers that contain bZIP1.

### Enriched Gene Ontology terms of the C/S1 dimer targets support their functional diversity

To gain insight into the potential biological functions of C/S1 bZIP homodimers and heterodimers resulting from DNA binding, we performed Gene Ontology (GO) enrichment analysis for the top 2000 target genes identified in DAP- and dDAP-seq based on their target scores (Fig. 3c). Overall, when we compared the top 2000 genes targeted by the S1 homodimers and the top 2000 genes targeted by S1:C heterodimers, about half of the genes (1108) were shared between the homodimers and heterodimers and the rest were unique (Fig. 3d). We observed that predicted targets of S1 and S1:C were enriched for high level biological processes consistent with known functions of this family, such as responses to abiotic stress and light, as well as carbohydrate metabolic processes^64^. Circadian rhythm, response to water and water deprivation, and response to hormones including auxin were enriched for almost all target gene sets (Fig. 3c Cluster 1, 2, and 4), suggesting these are the conserved functions of the S1 and C groups. For a subset of the heterodimers, multiple GO terms related to hypoxia response were strongly enriched (Fig. 3c Cluster 4). Hypoxia response is important in both stress tolerance and development. For instance, internal oxygen limitation occurs in the developing seed and could regulate seed growth and physiology^65^. Our results suggest that the important role of C/S1 bZIPs in seed development^33,58^ may be mediated by hypoxia-related target genes. In fact, the functions of bZIPs in tolerance to oxidative stress can be traced back as early as algae^66^. Since hypoxia responses are also relevant in the context of plant response to submergence^67,68^, we compared the S1 and S1:C targets to genes that are differentially expressed following submergence treatment^69^ and observed a much higher level of enrichment of S1 and S1:C targets in submergence-induced genes compared to submergence-repressed genes (Fig. 3e). Interestingly, we found the targets of bZIP11 homo- and heterodimers were highly enriched for responses to auxin (Fig. 3c box labeled a), consistent with the role of bZIP11 in modulating auxin-induced transcription^70^. Taken together, the target genes identified by DAP- and dDAP-seq recapitulated important aspects of C/S1 functions and could provide a baseline for studying how the functions of these TFs are regulated by additional processes.

Besides the common GO terms discussed above, target genes predicted for the S1 and S1:C pairs were strongly enriched in a diverse set of GO terms, showing a landscape of biological functions resulting from heterodimer DNA binding. For example, the GO terms related to responses to jasmonic acid, salicylic acid, and regulation of signaling were enriched in target genes of bZIP1:bZIP10 heterodimer but not in bZIP1:bZIP9 heterodimer (Fig. 3c boxes labeled b). bZIP44 homodimer target genes were significantly enriched in GO terms related to catabolic processes of cell wall components, which were not found for the bZIP44:bZIP10 heterodimer (Fig. 3c boxes labeled c). These data could be complemented by other molecular assays or higher order mutant analysis to investigate the role of heterodimerization in facilitating diverse biological functions of C/S1 bZIPs.

### Analysis of bZIP9 heterodimer targets revealed bZIP9 functions in ABA response

There is limited characterization of direct target genes of the group C bZIPs^32,34,38,55^ because heterodimerization is important for DNA binding, and it is challenging to investigate their biological functions due to a high degree of redundancy among members in the group. We hypothesized that dDAP-seq results for the S1:C bZIPs could help reveal the functions of C bZIPs and chose to explore this hypothesis by focusing on bZIP9.

**Fig. 4:**
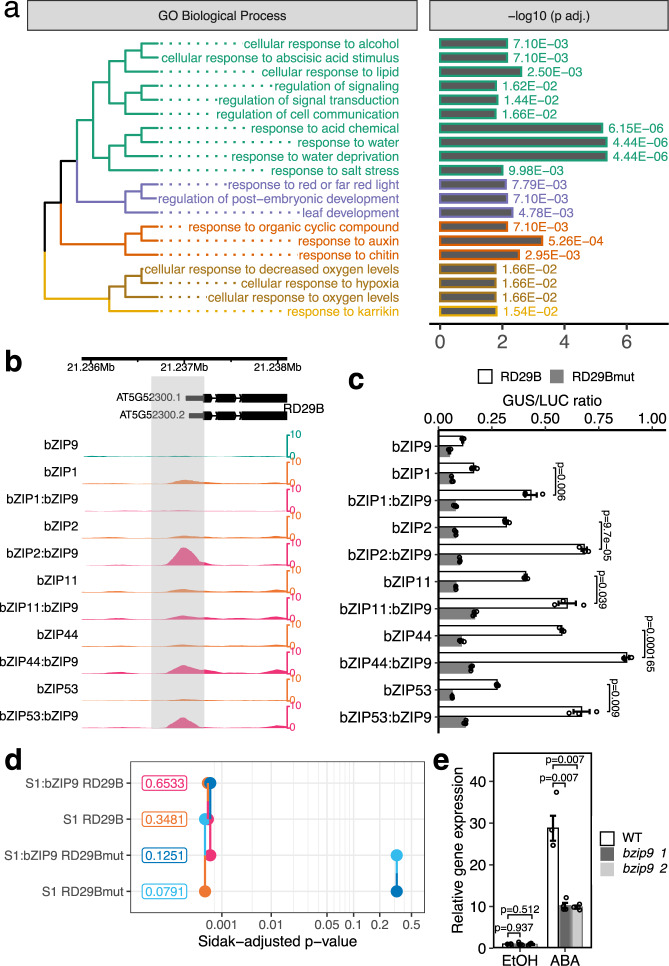
bZIP9 mediates ABA response. **a** Enriched GO terms for bZIPS1:bZIP9 heterodimer targets. dDAP-seq data of bZIP1:bZIP9, bZIP2:bZIP9, bZIP11:bZIP9, bZIP44:bZIP9, and bZIP53:bZIP9 heterodimers were combined to perform the GO enrichment analysis. *P*-values shown were computed by hypergeometric tests (one-sided Fisher exact tests) and corrected for multiple comparison by the Benjamini and Hochberg method. **b** bZIPS1:bZIP9 heterodimer showed stronger binding than S1 homodimers in the promoter region of ABA response marker genes *RD29B* (AT5G52310). Gray rectangle marks the 552 bp promoter region tested in the reporter assay. **c** Transient expression of bZIP9 with each bZIPS1 resulted in significantly higher expression of *RD29B::GUS* reporter compared to the bZIPS1 alone. RD29Bmut contained mutated sequences of three ACGTG elements and one TGAC half-site (Supplementary Data 6). *n* = 3 replicates. Data are normalized to luciferase control (LUC). Bar charts represent mean ± standard error (SE). *P*-values shown were computed by two-sided t-tests without adjustments for multiple comparisons. **d** Pairwise *P*-value plot^117^ comparing reporter activation in four combinations of effectors (S1 alone and S1 with bZIP9) and reporters (RD29B and RD29Bmut). The number next to each combination is the estimated mean activation averaged over S1 bZIPs. Each line segment connects a pair of combinations being compared, with point and half-line segment drawn for one combination in the color of the other combination in the pair. The x-axis location of the line segment indicates the *P*-value of the comparison. *P*-values were computed by two-sided tests comparing the estimated marginal means of the indicated factor combinations followed by Sidak correction for multiple comparisons. **e** Expression of *RD29B* genes in wild type (WT) and *bzip9* mutants after treatment of 50 µM ABA for 3 h with ethanol (EtOH) as negative control. Error bars indicate SE of three independent seedling pools. *P*-values shown were computed by two-sided *t*-tests without adjustments for multiple comparisons.

bZIP9 heterodimerizes with five S1 bZIPs^31,32,57^ and our dDAP-seq assay reported between 1774 and 28,658 peaks for the five heterodimers (Fig. 1b). GO analysis of the heterodimer target genes found a diverse set of functions for the individual heterodimers, including response to water, hypoxia, and auxin stimulus (Fig. 3c). We reasoned that the functions of bZIP9 could be identified by collectively analyzing the target genes of all its heterodimers. We called peaks by merging the dDAP-seq data from the five bZIP9 heterodimer experiments and calculated the enriched GO terms for the genes near the merged peaks. The highly enriched biological processes included response to abiotic stress such as water, salt and hypoxia, development, and response to hormones (Fig. 4a). One of the significantly enriched GO terms was response to abscisic acid (ABA), a plant hormone with well-characterized functions in response to water, salt, and post-embryonic development such as seed development^71–73^. We also found that the expression of *bZIP9* was one of the most strongly induced by ABA among the nine C/S1 *bZIPs* (Supplementary Figure 4a^74^). Therefore, we hypothesized that bZIP9 may be involved in the regulation of ABA response, which may contribute to the responses to stress and development.

To test our hypothesis, we started by examining bZIP9 heterodimer binding at known ABA response marker genes. *RD29A* and *RD29B* are two well-known markers of ABA response^75,76^, and we found strong binding signal at the promoters of these two genes by bZIP9 heterodimers with bZIP2, bZIP44, and bZIP53 (Fig. 4b and Supplementary Fig. 4b). Using a transient expression assay^77,78^ where expression of a GUS reporter was driven by promoters of *RD29A* and *RD29B* (*RD29Apro::GUS* and *RD29Bpro::GUS*), we found that co-transfection of bZIP9 with individual S1 bZIPs resulted in significantly higher reporter activation than transfections by each S1 bZIP alone (Fig. 4c and Supplementary Fig. 4c). For *RD29B*, the increase in reporter activation correlated with increase in binding of bZIP9 heterodimers (Fig. 4b, c) and the level of activation was reduced by mutating three ACGTG and one TGAC elements in the reporter construct. We fitted a linear model to analyze how changes in reporter activation by the presence of bZIP9 is dependent on the sequence motifs, and found the mutant sequence no longer produced significantly higher activation when comparing S1 and bZIP9 co-transfection to S1 transfection alone (Fig. 4d). For *RD29A*, the increase in reporter activation occurred without clear increase of heterodimer binding (Supplementary Fig. 4b, c) and mutating two ACGT elements in the reporter did not give significant changes in reporter activation regardless whether bZIP9 was present (Supplementary Fig. 4d), suggesting regulation by mechanisms beyond DNA binding. Furthermore, we used qPCR to check the expression of these two marker genes in two independent T-DNA mutant lines, *bzip9-1* and *bzip9-2* (Supplementary Fig. 4e–g), and found *RD29B* and *RD29A* expression was significantly reduced in the mutant compared to wild type following ABA treatment (Fig. 4e and Supplementary Fig. 4h). Therefore, the DNA binding and gene expression measurements support the role of bZIP9 in the regulation of ABA response, for which heterodimerization with S1 could be an important mechanism. In agreement, a C bZIP in wheat, TabZIP14-B, was reported to be a positive regulator of ABA and abiotic stress responses when expressed heterologously in *Arabidopsis*^79^, suggesting that the function of C bZIP in ABA response may be evolutionarily conserved.

### Distinct motifs underlie bZIP53 heterodimer DNA binding specificities

**Fig. 5:**
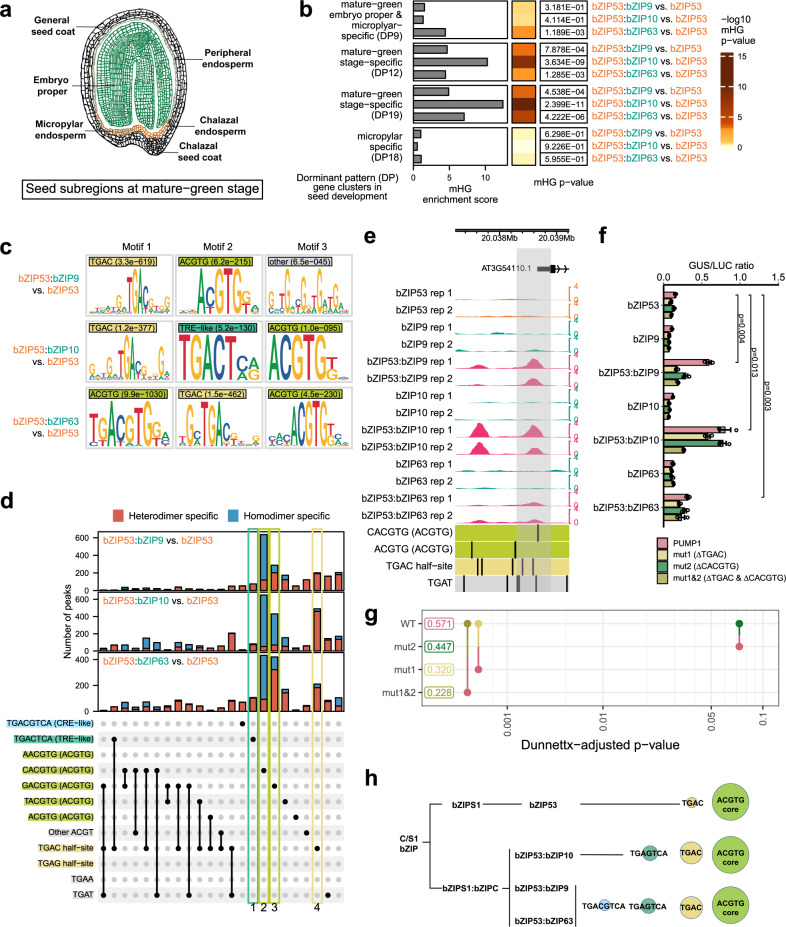
Target genes and motif differences revealed bZIP53:bZIPC heterodimer specificities. **a** Diagram of the subregions in a developing seed at the mature-green stage. Hand drawn on iPadOS using the Notes app. **b** Significance of association determined by mHG test between the genes differentially bound by bZIP53 heterodimers and subregion-specific gene expression at the mature-green stage in the developing seed. *P*-values shown were calculated by the mHG test for gene set enrichment without multiple comparison correction. **c** From sequences in the peaks specific to each bZIP53:bZIPC heterodimer, MEME discovered three major categories of enriched motifs: TGAC (marked by light yellow color), ACGTG (marked by pear color), and CRE-like element TGACTCA (marked by mint color). **d** KSM motif occurrences in peaks that showed increased binding comparing each bZIP53:bZIPC to bZIP53 (heterodimer-specific) or decreased binding (homodimer-specific). K-mer sequences corresponding to the motif categories in **c** are shown on the left. Each KSM motif instance in the peaks was checked for matches to the k-mer sequences, and the number of peaks containing the combinations of k-mer sequences are indicated by the bar plot at the top, separated into heterodimer- or homodimer-specific peaks. The category of sequence elements for each k-mer is included in parentheses and indicated by the background color as in **c**. **e** Two bZIP53:bZIPC heterodimer-specific peaks upstream of *PUMP1* (AT3G54110). Gray rectangle marks the 582 bp promoter region tested in the reporter assay. **f** Transient expression of bZIP53 with each C bZIP resulted in significant increase in expression of the *PUMP1::GUS* reporter that was reduced by mutating the TGAC half-site (mut1), the ACGTG element (mut2), or both (mut1&2) (Supplementary Data 6). *n* = 3 replicates. Data are normalized to luciferase control (LUC). Bar charts represent mean ± standard error (SE). *P*-values shown were computed by two-sided t-tests without adjustments for multiple comparisons. **g** Pairwise p-value plot^117^ comparing reporter activation of mutated reporters to wild-type (WT) in bZIP53:bZIPC. The number next to each reporter is the estimated mean activation averaged over bZIP53:bZIPC experiments. Each line segment connects a pair of reporters being compared, with point and half-line segment drawn for one reporter in the color of the other reporter in the pair. The *x*-axis location of the line segment indicates the *P*-value of the comparison. *P*-values were computed by two-sided tests comparing the estimated marginal means of the indicated factor combinations followed by Dunnettx correction for multiple comparisons. **h** Model of DNA sequence specificity of bZIP53 homodimer and bZIP53:bZIPC heterodimers.

The function of bZIP53 (group S1) in regulating seed maturation has been extensively characterized^33,58^, and its heterodimerization with group C bZIPs was shown to be an important mechanism for regulating a handful of target genes. To systematically investigate the contributions of bZIP53 heterodimers to seed development, we first identified heterodimer-specific binding sites by performing differential binding analysis comparing the bZIP53 heterodimer experiments to the bZIP53 homodimer experiment (for example, bZIP53:bZIP9 to bZIP53, bZIP53:bZIP10 to bZIP53). From each pairwise comparison, we obtained between 2143 and 9136 peaks that were more enriched in the heterodimer compared to the homodimer (heterodimer-specific) and between 851 and 1817 peaks that were less enriched (homodimer-specific) using adjusted *P*-value threshold of 0.05 (Supplementary Data 3). We then compared the genes near the differentially bound peaks to those expressed in specific seed sub-regions or during specific stages in the developing seed^80^. Of the 47 sets of genes showing dominant patterns (DP) of expression in specific subregions and stages in the developing seed, the differentially bound targets for bZIP53 heterodimers were highly enriched for genes in DP9, DP12, and DP19, all three of which are specifically expressed at the mature green stage in multiple seed subregions (Fig. 5a, b and Supplementary Fig. 5a). In contrast, we did not observe enrichment for genes showing other dominant expression patterns, such as DP18 that are expressed specifically in the micropylar (Fig. 5b and Supplementary Fig. 5a). These results provide direct evidence for the role of DNA binding by bZIP53 heterodimers in regulating gene expression during seed maturation. Consistent with the finding that heterodimerization with bZIP10 increases the DNA binding affinity of bZIP53 at the promoter of seed maturation genes such as *2S2*^33,58^, we observed that the binding of bZIP53 to several seed maturation genes, such as *2S1*, *2S2*, and *CRU3*, was dependent on heterodimerization with bZIP10 (Supplementary Fig. 5b, c). Therefore, bZIP53 heterodimer-specific binding events are associated with a subset of genes that have a unique spatial-temporal pattern of expression in the developing seed, illustrating the power of using dDAP-seq to investigate the biological processes regulated by S1:C heterodimers. Although enrichment for seed maturation genes was also observed for DNA binding targets of bZIP10 heterodimer with bZIP1, the other S1 bZIP expressed in seeds (Supplementary Fig. 5a), the role of bZIP1 in seed maturation was previously excluded based on its inability to activate a handful of seed maturation genes^46^. Future studies could examine whether bZIP1:bZIP10 heterodimer could activate other seed maturation genes and whether mechanisms beyond DNA binding exclude bZIP1 from regulating seed maturation.

To better understand the sequence basis underlying the differential binding by homodimers and heterodimers, we sought to identify sequence motifs enriched in the homodimer- and heterodimer-specific binding events. To do this, we first extracted sequences under the peaks that are specific to each of the bZIP53:bZIPC heterodimer when compared to the bZIP53 homodimer (2000 peaks with highest fold change in binding and adjusted *P*-value ≤ 0.05), and used these sets of sequences for de novo motif discovery by MEME^81^. The top three motifs enriched in the bZIP53 heterodimer-specific peaks fell into three major categories (Fig. 5c): a motif that contained a TGAC central sequence, a motif that contained a core ACGTG sequence, and a motif that contained the sequence TGACTCA. We noted that the first two motifs were similar to the motifs in Fig. 2c identified from the most enriched 1000 peaks for each dimer, however the differential analysis uncovered additional motifs that distinguished between hetero- and homodimers. While the motif containing the core ACGTG sequence corresponded to two well-known ACGT *cis*-elements in plants, including the G-box (CACGTG) and GC-hybrid (GACGTG) sequences^23^, the TGACTCA motif overlapped with the ACT element (ACTCAT/ATGAGT) that was previously identified for a bZIP53 heterodimer at a handful of target sites^27,28,35^. Binding at motifs containing a TGAC sequence that is not part of an ACGT element has not been reported for plant bZIPs. The 2000 peaks with the largest increase in binding comparing the bZIP53:bZIP10 heterodimer to the bZIP53 homodimer contained 147 instances of the TGACTCA motif and 1475 instances of the TGAC motif that did not contain TGACTCA or ACGT, suggesting these non-ACGT elements could make a major contribution to DNA target recognition by the heterodimers. In fact, these two non-ACGT elements correspond to the high affinity binding sites previously identified for the yeast bZIP GCN4: the TRE-like element TGACTCA and the half-site TGAC. GCN4 further recognizes a third motif, the CRE-like element TGACGTCA^42–44^, which was not found among the PWM motifs enriched in bZIP53:bZIP10 heterodimer-specific binding but are strongly preferred by group D bZIPs^47,82–86^. The presence of the relatively distinct motif categories motivated us to perform a higher-resolution, k-mer-based analysis to better capture the complex sequence specificities that may not be completely represented by PWM models used by MEME^87–89^.

From the major motif categories describe above, we determined the k-mer sequences that gave rise to each category and investigated the occurrences of these k-mer sequences in motif matches contained in the heterodimer- and homodimer-specific binding events (Fig. 5d and Supplementary Data 4). These k-mer sequences included the CRE-like elements, TRE-like elements, four possible ACGTG elements varying at the position before the ACGT, and 4-mer sequences in the PWM models that were not part of the previous categories: TGAC half-site (TGAC that is not part of CRE-like elements), TGAG half-site (TGAG that is not part of TRE-like elements) and TGAT. For finding motif matches, we used the k-mer set memory (KSM) motif representation and matching calculation^90^. Each KSM model comprise a set of aligned k-mers overrepresented in TF binding sites, capable of preserving positional dependencies and proximal flanking bases. KSM motifs were shown to more accurately model TF recognition sequences than PWM and more complex methods that incorporated high degree of positional dependencies^90^. For the three bZIP53 heterodimers, most peaks containing the TGACTCA or TGAC half-site KSM matches were heterodimer specific (Fig. 5d Box 1 and Box 4; Supplementary Data 4). KSM matches that contained the G-box element CACGTG were found in much higher frequency in homodimer-specific peaks (Fig. 5d Box 2). Interestingly, while bZIP53:bZIP10 was more associated with the homodimer-specific peaks that contained the GC-hybrid (GACGTG) than the heterodimer-specific peaks, the reverse was true for bZIP53:bZIP9 and bZIP53:bZIP63 (Fig. 5d Box 3). This information could not be readily obtained by inspecting the ACGTG PWMs reported by MEME (Fig. 5c), suggesting that our k-mer-based analysis was more sensitive in finding sequences targeted by homodimer- or heterodimer-specific binding. Figure 5e shows two examples of heterodimer-specific peaks in the upstream and promoter region of *PUMP1* (plant uncoupling mitochondrial protein 1) and the motif instances in the peaks. The peak closest to the TSS contains a TGAC half-site and a ACGTG element near the peak summit. In a reporter assay using 500 bp sequence upstream from TSS that contains this peak, reporter activation was higher when bZIP53 was co-transfected with each of C bZIPs, bZIP9, bZIP10, and bZIP63, than transfection by bZIP53 alone (Fig. 5f). Mutating the TGAC half-site (mut1) resulted in stronger reduction in activation compared to mutating the ACGTG sequence (mut2) (Fig. 5g). The mutations did not return activation to basal level, likely due to the presence of additional elements present in the 500 bp promoter sequence. The peak further upstream contains two TGAC half-sites at the peak summit, but no ACGTG elements that are preferred by bZIP53 homodimers.

Based on these results, we propose a model for the DNA binding specificities of bZIP53 homodimer and heterodimers (Fig. 5h). In general, bZIP53 homodimer mainly recognizes the classic ACGTG core motif and the TGAC half-site that is also recognized by GCN4. The bZIP53:bZIP10 heterodimer binds two types of GCN4-like motifs, the TGAC half-site and the TRE-like element TGACTCA. In addition to these motifs, bZIP53:bZIP9 and bZIP53:bZIP63 recognize a CRE-like element TGACGTCA just like GCN4. These sequence specificities provide a molecular basis for the overlapping and expanded target genes of bZIP53 heterodimers that could potentially be a general mechanism mediating the dynamic functions of C/S1 bZIPs.

### General patterns of altered DNA binding specificities by C/S1 dimerization

**Fig. 6:**
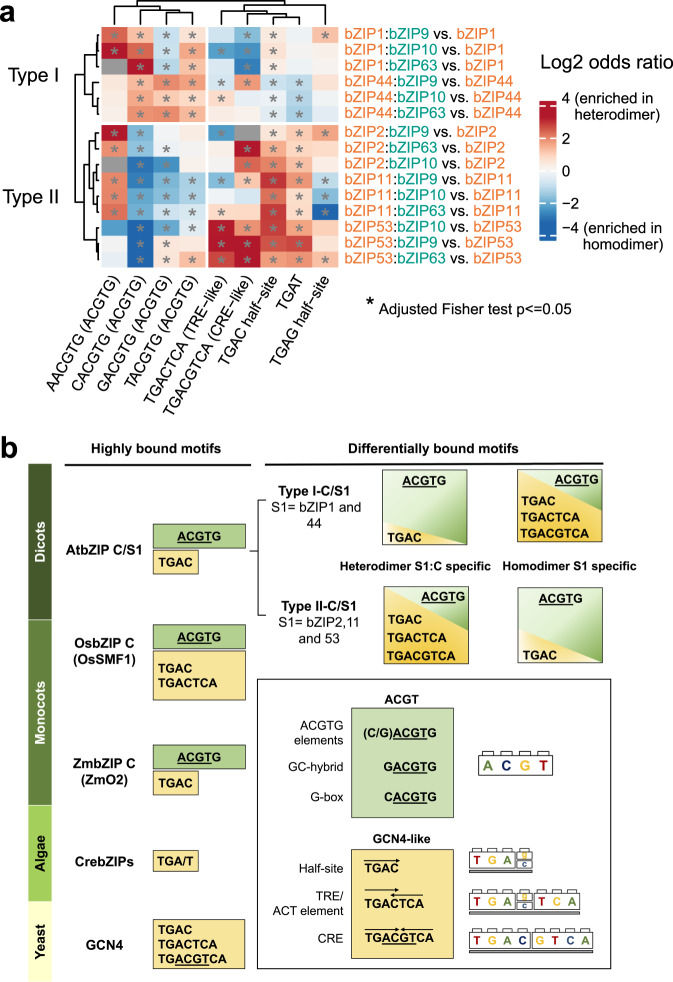
Distinct patterns of DNA binding specificities of bZIP C/S1 dimers. **a** Relative motif enrichment of differentially bound peaks for S1 homodimers and S1:C heterodimers. Odds ratios and *P*-values were computed by one-tailed Fisher exact and P-values were adjusted for multiple comparison by the Benjamini and Hochberg method. **b** Summary of DNA binding specificities of selected bZIPs in yeast and algae, and selected C/S1 bZIPs in monocot and dicot plant species, showing the yeast GCN4-like motifs and the ACGT elements previously characterized in plants (supporting data in Supplementary Fig. 6). Dimers of *Arabidopsis* C/S1 bZIPs have two types of sequence specificities determined by the relative enrichment of the GCN4-like motifs and the ACGT elements.

To investigate whether the sequence patterns identified for bZIP53 heterodimer-specific binding could be generalized to other S1 bZIPs, we calculated the relative enrichment of these patterns in heterodimer-specific vs. homodimer-specific peaks obtained by comparing the S1:C dDAP-seq data to the corresponding S1 DAP-seq data (Fig. 6a, Supplementary Fig. 5d, and Supplementary Data 4). More than half of the heterodimer-specific peaks are located within 1 kb of the TSS (Supplementary Fig. 5e). We observed two types of motif enrichment patterns: Type I consists of dimers of bZIP1 and bZIP44 and Type II consists of dimers of bZIP2, bZIP11 and bZIP53. For Type I, the homodimers prefer the GCN4-like motifs including the TGAC half-site, the TRE-like element TGACTCA and the CRE-like element TGACGTCA, while heterodimers prefer the ACGTG elements. Interestingly, Type II motif enrichment shows the opposite trend, where the heterodimers prefer the GCN4-like motifs and homodimers prefer the classic ACGTG elements. Therefore, although the ACGTG elements are the most enriched motifs for all the C/S1 homodimers and heterodimers (Fig. 2c) and the genome-wide binding profiles are highly similar between all the dimers (Fig. 2b), there is heterodimer-specific binding that could be explained by sequence elements that correspond to the set of sequences recognized by the yeast bZIP GCN4.

## Discussion

TF interactions are key to combinatorial regulation of gene expression, so it is important to understand how interacting TFs achieve functional diversities and specificities. In this study, we upgraded the DAP-seq methodology suite to incorporate double DAP-seq, which allows mapping of genome-wide binding sites of interacting TFs in an endogenous genomic context. By expressing one or two TFs in vitro and comparing their bound sequences on genomic DNA, we could directly identify how TF interactions modulate binding specificities, potential target genes in the genome and biological functions. dDAP-seq is straightforward to carry out, easy to scale up, and identifies highly accurate motifs and binding events in the whole genome. Previous studies that used synthetic oligonucleotides to study binding by heterodimers across TF families and major groups of the bZIP family in human uncovered rules governing heterodimer binding in terms of changes in motif sequence and spacing constraints^10,11,91^. For the two closely related bZIP groups C and S1, the primary motifs targeted by the homo- and heterodimers are highly similar (Fig. 2c). However, since the DNA library in dDAP-seq contained the sequence diversity and binding site context of the genome, we were able to uncover the differences in sequence preference between homo- and heterodimers. This system can be used to interrogate TF pairs or higher order complexes where interaction is required for DNA binding. For instance, the MADS-box TFs bind DNA as obligate dimers^92,93^, which could be tested in dDAP-seq. On the other hand, if two TFs can produce DAP-seq peaks individually, the dDAP-seq assay could be easily adjusted to allow affinity purification using the two tags sequentially to investigate the effect of interaction^94^, such as the heterodimerization between S1 bZIPs and between C/S1, G, and H bZIPs^32^.

In this study, we demonstrated the sensitivity of dDAP-seq to delineate the overlapping and specific DNA binding properties between the closely related C/S1 bZIPs. While group C bZIPs bind to a handful of DNA sequences in in vitro binding assays such as EMSA^38,55,56,58^, they did not produce peaks in DAP-seq. This discrepancy may be explained by differences in the experimental conditions, including protein expression and purification and the nature of DNA input^57^. It is also possible that group C binding to these sites have low affinity or dissociate easily in the DAP-seq conditions, and dimerization with S1 in dDAP-seq increases the binding stability to produce peaks, consistent with previous findings that S1:C heterodimer has higher affinity than S1 homodimers at some targets^32,33,38^. The DNA binding differences identified by comparing dDAP-seq and DAP-seq are correlated with heterodimer-induced reporter activation for *2S2* (ref. ^33^ and Supplementary Fig, 5b), *RD29B* (Fig. 4b, c), and *PUMP1* (Fig. 5e, f), suggesting DNA binding differences of heterodimers could lead to transcriptional changes. For *RD29A*, heterodimer-specific activation occurred without changes in DNA binding (Supplementary Fig. 4b, c), suggesting it could be regulated by mechanism beyond DNA binding, such as recruitment of other factors or activation machinery^95–97^. Our DAP- and dDAP-seq dataset provided a baseline in terms of genome-wide DNA binding for elucidating the functions of these heterodimers, which are subject to additional complex regulation including transcription, uORF regulation, post-translational modification of the TFs^95,97^ and chromatin accessibility of the binding sites. Since dDAP-seq does not directly show dimerization state at the enriched regions and the transcriptional effects of binding, for selected binding sites of interest EMSA and reporter assays can be used to confirm dimerization and evaluate transcriptional effect, respectively.

Evolutionary adaptation to various environmental stimuli has generally coincided with an increase in gene copy number and complexity of gene regulation^98^. Specifically, for the bZIP family that has undergone parallel expansion in plant and animal lineages, dimerization between family members is a critical mechanism to expand the DNA targeting repertoire and enable regulatory complexity and functional diversification^11,99^. Group C bZIPs in plants expanded from a Proto-C bZIP present early in the green algae Chlorophyta^100,101^. Group S1 likely originated from Proto-C or group C by gene duplication, possessing similar protein structures to form dimers with the group C bZIPs. The C/S1 dimers are postulated to first appear in the multicellular green algae Charophyta^100,101^. C/S1 dimerization is therefore a deeply conserved mechanism and is expected to regulate conserved target genes and biological processes. Our GO enrichment analysis of target genes for C/S1 dimers based exclusively on DNA binding revealed biological processes correlated with two major evolutionary events in the history of plant evolution^102^. First, the processes related to response to biotic and abiotic stress, circadian rhythm, phytohormone signaling are ancient functions underlying the development of multicellularity. A second set of GO terms, related to leaf development and response to water and salt, are functions required for land colonization. Subsequent evolution of seed plants involved major expansion of seed genes^102^. The regulation of seed-specific gene expression could be a more recent function carried out by the heterodimers. Beyond C/S1, the major sub-clades of bZIPs have a different set of specific functions, for example, group D functions in biotic response and group I in development^20,49^. We expect dDAP-seq could be readily applied to study the DNA binding specificity of heterodimers involving these bZIPs.

In Fig. 6b, we summarized the two types of motif enrichment patterns for the C/S1 dimers along with the DNA binding specificities of GCN4 in yeast, selected bZIPs in algae and C/S1 bZIPs in monocot and dicot species based on available data (Supplementary Fig. 6). The three motifs recognized by GCN4, the CRE-like element TGACGTCA, the TRE-like element TGACTCA and the TGAC half-site^43,103–107^, may reflect the targets of an ancestral bZIP family in the unicellular common ancestor of fungi, plant and metazoan. The ACGTG elements are similar to the CRE-like element and were derived from this common ancestor^16^. In *Arabidopsis*, group D bZIP recognizes the CRE-like sequence TGACGTCA, while group S and group A bZIPs recognize the ACGTG element. The TGAC half-site we found for C/S1 dimers in this study has not been reported for any *Arabidopsis* bZIPs, although it is part of the consensus binding site GGATGAC identified using an in vitro expressed group C bZIP from rice^29,108^, suggesting this recognition site could be conserved in higher plants. The ability of C/S1 dimers to recognize both the TGAC half-site and the ACGTG elements may mediate their distinct biological functions compared to the other bZIP groups. Furthermore, within the group C/S1, homodimer and heterodimer binding are distinguished by their relative preference for the GCN4-like and ACGTG elements, reflecting the increased DNA binding flexibility conferred by dimerization. Future DAP- or dDAP-seq experiments with C/S1 bZIPs in targeted species in the green lineage^29,109,110^ could shed light on the evolution of DNA binding specificity of this group.

The DNA binding domains of the bZIP TFs are highly conserved between plants and animals^111^, and many important insights of protein-DNA interaction have been obtained by studying this family. For plant bZIPs, the sequences flanking the ACGT elements have been extensively investigated for their role in determining DNA binding activity^23,112^. Recent analyses of published DAP-seq data, focused on the core ACGT motif, showed that the flanking sequences were insufficient to determine binding^112^ and that DNA shape surrounding the core motif contributed to differential binding by family members^113^. For human bZIPs, PBM experiments found that DNA binding specificities of the heterodimers were grouped into three major classes in relation to the homodimer binding sites: juxtaposing homodimer half-sites, overlapping homodimer half-sites, and emergent sites not readily inferred from the homodimer half-sites^11^. While some heterodimers bind to multiple classes of cognate sites, most display highly complex specificity landscapes that defy simple characterization. In this study, by investigating binding patterns on endogenous DNA that contains the sequence diversity and context of the thousands of binding sites in real genomes, we found that the dimers between the closely related C/S1 subgroups were distinguished by differential preference for two sets of motifs related to the GCN4 recognition sequences and ACGTG elements. As we showed previously^47^, assaying TF binding directly on genomic DNA fragments could capture the genomic properties that influence TF binding in vivo. It is therefore worthwhile to investigate whether general principles of DNA binding specificity of bZIP heterodimers may emerge on real genomic DNA from the complex specificity landscape determined on synthetic oligonucleotides. This knowledge is critical for more accurate prediction of how trait- or disease-associated genetic variants could impact DNA binding and combinatorial gene regulation.

## Methods

### Plant materials, treatments, and phenotypic assays

For DAP-seq and dDAP-seq DNA libraires, *Arabidopsis* reference accession Col-0 (CS70000) was grown in soil at 22 °C under long-day (16 h light/8 h dark) conditions for three weeks. Rosette leaves were collected and flash frozen with liquid nitrogen prior to genomic DNA isolation. For ABA treatment experiments, seeds of Col-0 (WT) and *bzip9* mutants (*bzip9-1*/SALK_093416 and *bzip9-2*/SAIL_569_C12) were surface sterilized by 50% bleach containing 0.05% Triton-X100 for 10 min and washed three times by sterilized water. After 3 days of stratification at 4 °C, seeds were grown for 7 days at 22 °C in long day conditions on plates containing 1 x Linsmaier and Skoog (LS) medium, pH 5.7 (Caisson laboratories, LSP03-1LT) with 0.8% agar (Caisson laboratories, A038-500GM), and 10 mg/ml sucrose (Fisher Scientific, 57-50-1). For ABA treatment, 7-day-old seedlings were transferred into the liquid LS medium with 50 μM (±) Abscisic Acid (ABA; PhytoTech Labs, A102) dissolved in ethanol (EtOH; 200 Proof, Pharmco, 100135). The same amounts of ethanol in the liquid LS medium were used as control. After 3 h treatment in ABA or EtOH, the seedlings were immediately frozen in liquid nitrogen for RNA extraction.

### RNA extraction and cDNA synthesis

Total RNA was extracted by using RNeasy Mini Kit (Qiagen, 74104). RNA concentrations and 260/280 nm ratios were determined with a NanoDrop 2000. Total RNA was treated with RNase-Free DNase Set (Qiagen, 79254) to remove the contaminating DNA. Two μg of RNA was used for cDNA synthesis with SuperScript™ III First-Strand Synthesis System (Invitrogen, 18080051). The diluted cDNA was used as the template for quantitative real-time PCR (qRT-PCR) analysis using Roche 480 LightCycler. The primers used are listed in the Supplementary Data 5.

### Standard DAP-seq and double DAP-seq experiments

Genomic DNA libraries preparation: The DAP- and dDAP-seq genomic DNA (gDNA) library was prepared as a standard high throughput gDNA sequencing library for the Illumina platform. Five mg gDNA eluted in 130 μl Elution buffer (10 mM Tris-Cl, pH 8.5) was fragmented to an average of 200 bp using Covaris S220 Sonicator, with protocol setup “Peak Power 175.0, Duty Factor 10.0, Cycle/Burst 200, Duration 180 s, Sample temperature 4–8 °C”. The reaction was precipitated by 2x volume 100% cold ethanol, washed by 70% cold ethanol and was suspended in 34 μl Elution buffer. The fragmented gDNA was end-repaired in 50 μl reaction using the End-It DNA End-Repair Kit (Lucigen, ER81050), incubated at room temperature for 45 min. The reaction was precipitated by 2x volume 100% cold ethanol, washed by 70% cold ethanol and was suspended in 32 μl Elution buffer and used in a 50 μl A-tailing reaction using Klenow (3′→5′ exo-) (NEB, M0212), incubated at 37 °C for 30 min. The reaction was precipitated by 2x volume 100% cold ethanol, washed by 70% cold ethanol and was suspended in 30 μl Elution buffer before adapter ligation. A-tailed gDNA fragments and 10 μl 30 μM annealed adapters were ligated in a 50 μl ligation reaction using T4 DNA Ligase (Promega, M1804) incubated at room temperature for 3 h. The DNA was precipitated and suspended in 30 μl Elution Buffer as the gDNA libraries used in DAP-seq and dDAP-seq.

TF protein preparation: The *pIX-Halo-bZIP* and empty *pIX-Halo* plasmids were gifts from Joseph Ecker lab. The *pIX-SBP::ccdB-CAM*^R^ vector was generated by ligating a double stranded oligo containing the SBP-Tag sequence^10,114^ into the EcoRV and SacI sites of a *pIX::ccdB-CAM*^R^ vector backbone and transforming into ccdB survival2 cells (ThermoFisher). To create the *pIX-SBP-bZIP* expression plasmids, *pEntry-bZIP-ORFs* (also gifts from Ecker lab) were recombined using LR clonase (Gateway™ LR Clonase™ II Enzyme mix, ThermoFisher, 11791020) into the *pIX-SBP* vector (gifted by Mary Galli, Gallavotti lab). Plasmid DNA was extracted using the E.Z.N.A. Plasmid DNA Mini Kit I (Omega Bio-tek, D6942-01). *pIX-Halo-bZIPs* (N terminal Halo fusion tag: Halo-bZIP1, 2, 11, 44, 53, 9, 10, 25, and 63) and *pIX-SBP-bZIPs* (N terminal SBP fusion tag: SBP-bZIP1, 2, 11, 44, 53, 9, 10, 25, and 63) were expressed using the TNT SP6 Coupled Reticulocyte Lysate System (Promega, L4600). Expression of Halo-bZIP and SBP-bZIP proteins were confirmed by western blotting using anti-HaloTag monoclonal antibody (Promega, G9211) and anti-SBPTag mouse anti-human antibody (MilliporeSigma, MAB10764).

Pulldown and western blot experiments for protein-protein interaction: In vitro expressed Halo-protein and SBP-protein were incubated with Halo beads in 100 μl wash buffer overnight on a rotator at 4 °C. The beads were washed with 100 μl cold wash buffer (PBS + 0.05% NP40) for five times to remove non-binding proteins. The protein complex was eluted by heating the beads at 98 °C. The SBPTag mouse anti-human antibody (MilliporeSigma, MAB10764) was used to detect the presence of SBPTag-protein in the supernatant.

Standard DNA affinity purification sequencing (DAP-seq): Fifty μl protein expression reaction using TNT SP6 Coupled Reticulocyte Lysate System (Promega, L4600) containing 1000 ng *pIX-HALO-bZIP* plasmids was incubated for 3 h at 30 °C. The reaction was then mixed with 10–20 μl Magne HaloTag Beads (Promega, G7282) and 50 μl wash buffer (PBS + 0.05% NP40) on a rotator for 1 h at room temperature. The beads with protein were then washed five times on a magnet with 100 μl wash buffer to purify HaloTag-fused protein. The protein-bound beads were incubated with 100 ng adapter-ligated gDNA library in 100 μl wash buffer for 2 h. The beads were then washed 5 times with wash buffer to remove unbound ligated DNA fragments. The beads were suspended in 30 μl elution buffer, heated at 98 °C for 10 min, and put on ice immediately for 5 min to denature the protein and release the bound DNA fragments. 25 μl of the supernatant was used for the PCR enrichment step.

Double DNA affinity purification sequencing (dDAP-seq): One hundred μl protein expression reaction of TNT SP6 Coupled Reticulocyte Lysate System (Promega, L4600) containing 1000 ng *pIX-HALO-bZIP* plasmids and 1500 ng *pIX-SBP-bZIP* plasmids were incubated for 3 h at 30 °C. The reaction was mixed with 10–20 μl Magne HaloTag beads, 100 μl wash buffer and 100 ng adapter-ligated DNA on a rotator overnight at 4 °C. Halo beads were then washed five times on a magnet with wash buffer to purify the HaloTag-fused protein with its interacting partner. 100 ng adapter-ligated gDNA libraries and 100 μl wash buffer were added to the reaction and incubated for 6–8 h at 4 °C. The beads were washed and suspended with 30 μl elution buffer, heated at 98 °C for 10 min, and put on ice immediately for 5 min to denature the protein and release the bound DNA fragments. 25 μl of the supernatant was used for the PCR enrichment step.

Sequential DAP-seq: SBPTag-fused bZIP and HaloTag-fused bZIP were co-expressed as in the dDAP-seq. The co-expressed proteins were incubated with 300 ng of adapter-ligated DNA for 1 h at room temperature; 1.5 μl of pre-blocked Streptavidin magnetic beads were then added to the sample and rotated at room temperature for 2 h. Beads were washed seven times in wash buffer and TF-DNA complexes were eluted with 10 μM biotin. In all, 50 μl of elute was then incubated with 10 μl of HALO beads and rotated for 1 h at room temperature. Halo beads were washed ten times in wash buffer. DNA was eluted from the beads by heating at 98 °C for 10 min and put on ice immediately for 5 min to denature the protein and release the bound DNA fragments. 25 μl of the supernatant was used for the PCR enrichment step.

Amplification of the DAP- and dDAP-seq library and sample pooling: The PCR reactions were prepared as follows: Mix 1 μl of Phusion DNA Polymerase (New England Biolabs, M0530), 10 μl of 5x Phusion HF Buffer, 2.5 μl of 10 mM dNTPs, 1 μl of Primer A (25 μM) and 1 μl of Primer B (25 μM), 25 μl of eluted DNA, add water to 50 μl. The Primer A and Primer B sequences contain unique indexes^115^ for each sample to be pooled in one sequencing run. The eluted DNA was amplified with the following PCR conditions: 98 °C for 2 min, 15–19 cycles of 98 °C for 15 s, 60 °C for 30 s, and 72 °C for 1–2 min, final extension at 72 °C for 10 min. The samples were pooled and run in 1% agarose gel. The gel was cut to purify fragments from 200 bp to 600 bp using Zymoclean Gel DNA Recovery Kit (Zymo Research, D4007). The purified DNA libraries were measured by the Qubit HS ds DNA Assay Kit (ThermoFisher, Q32854) and sequenced on the Illumina platform. Relevant primer sequences are listed in the Supplementary Data 5.

### Reporter assay in protoplasts

To generate the effector plasmids used in reporter assay, the full-length CDSs of *bZIP9*, *bZIP1*, *bZIP2*, *bZIP11*, *bZIP44*, and *bZIP53* were cloned in *pUC19-35S-DC* using LR reactions. Approximately 500 bp promoter fragments and the motif mutated versions of *RD29A*, *RD29B*, and *PUMP1* genes containing DAP- or dDAP-seq peaks were synthesized by Twist BioScience based on *Arabidopsis* wild-type Col-0 genome. Sequences are shown in Supplementary Data 6. All the promoter region fragments are subcloned into *pUC19-DC-GUS* as reporter plasmids. The combinations of effector plasmids, reporter plasmids, and reference plasmids (*pUC19-35S-LUC*) were transformed into *Arabidopsis* protoplasts^78^. Briefly, the middle section of four-week-old fully expanded leaves were cut out, sliced into strips and immersed into enzyme solution containing 0.4 M mannitol, 20 mM KCl, 20 mM MES, 10 mM CaCl_2_, 5 mM β-mercaptoethanol, 0.1% BSA, 0.4% macerozyme R10, and 1.5% cellulase R10. The mixture was incubated at room temperature for 2 h before filtering the protoplasts through a 75 μm nylon mesh and washing them with W5 solution (154 mM NaCl, 125 mM CaCl_2_, 5 mM KCl, and 2 mM MES). After centrifuging at 1000 rpm for 3 min at 4 °C, the protoplasts were resuspended with MMg solution (0.8 M mannitol, 1 M MgCl_2_ and 0.2 M MES) to obtain a concentration of 200,000 cells per ml. Next, 6 μg effector, and 3 μg reporter and 100 ng reference plasmids were co-transfected into 100 μl of protoplasts using the PEG-calcium mediated transfection method, followed by incubation in darkness for 18 to 20 h at room temperature. The GUS activity assay was conducted as described in Tiwari et al. 2003^77^ and measured using a Fluoroskan microplate reader. The MUG (4-Methylumbelliferyl β-d-glucuronide) (Sigma-Aldrich, M9130) and luciferase assay system (Promega, E1500) were used to perform GUS and LUC activity assays, respectively. Relative GUS activity was calculated via normalization to LUC activity, and the data are presented as three independent biological replicates. Relevant primer sequences are listed in the Supplementary Data 5.

### Linear models and post hoc comparisons

For each of the *RD29A* and *RD29B* reporter assays, a linear model is fitted to the formula *response* ~ construct*c + *s1*, where *response* is the reporter activation normalized to LUC, *construct* is wild-type or mutant sequence, *c* is the presence or absence of bZIP9, and *s1* is one of bZIP1, bZIP11, bZIP44, bZIP53. For PUMP1, a linear model is fitted to the formula *response* ~ *construct* + *c* for the bZIP53:bZIPC experiments, where *response* is the reporter activation normalized to LUC, *construct* is one of WT, mut1, mut2, and mut1&2, and *c* is one of bZIP9, bZIP10, and bZIP63. The models were checked by DHARMa^116^ (version 0.4.6) for uniformity and dispersion. The package emmeans^117^ (version 1.7.5) was used to perform post hoc comparisons and create the pairwise *P*-value plots with the indicated contrasts and *P*-value adjustment methods.

### DAP-seq and dDAP-seq data processing and analysis

Read processing, normalization and peak calling: The DAP-seq and dDAP-seq libraries were sequenced on an Illumina NextSeq 500. Adapter sequences were trimmed from the reads in the FASTQ files by Trim Galore (version 0.6.6) and Cutadapt version 3.1 with quality cutoff of 20^118^. The trimmed the reads were mapped to the *Arabidopsis* reference genome sequence TAIR10 using Bowtie2^119^ (version 2.2.9) with default parameters. Aligned reads were filtered by mapping quality score of at least 30. Peak calling was done by the GEM peak caller^120^ (version 3.3) on the filtered mapped reads with the default read distribution, TAIR10 nuclear chromosome sequences, q-value threshold of 0.01, and parameters “--f SAM ---t 1 --k_min 5 --k_max 14 --k_seqs 2000 --k_neg_dinu_shuffle --outNP --outBED --outMEME --outJASPAR --outHOMER --print_bound_seqs --print_aligned_seqs”. Peaks were called for each replicate individually or by merging the two replicates using GEM’s multi-replicate mode, with samples from experiments of empty vector *pIXHALO* and/or *pIXSBP* as control. Combined peaks for bZIP9 were called using reads from the five heterodimer dDAP-seq experiments by the GEM multi-condition mode. Each GEM run created two set of binding event calls: GEM events that were optimized for both read enrichment and centrally located motifs, and GPS events that were optimized for read enrichment only. To create the blacklist regions that contain highly enriched but artifact signals, peak calling was done for a set of negative control samples using MACS3^121^, which could find broader regions of read enrichment compared to the point-source binding events reported by GEM. The set of control samples included 18 experiments with replicates where sequence-specific DNA binding was not expected to occur: DAP-seq of *pIXHALO* empty vector by HaloTag beads, DAP-seq of *pIXSBP* empty vector by SBPTag beads, double DAP-seq of *pIXHALO* empty vector and SBPTag-fused bZIP S1 by HaloTag beads, and double DAP-seq of HaloTag-fused bZIP C and empty vector *pIXSBP* by HaloTag beads. The peaks reported by MACS3 (version 3.0) for these control samples were merged and the peak regions shared by at least 5 of these control samples were used as blacklist in downstream analysis. Merged peak sets for bZIP groups were created by the program muMerge for peak overlap or target enrichment analysis^122^.

BigWig files of normalized read signals were created using the MAPQ 30 filtered alignment BAM files by the bamCoverage program in the deepTools package^123^ (version 3.5.0) with the following parameters: “--binSize 1 --normalizeUsing RPKM --ignoreForNormalization Mt Pt”. The genome browser tracks were plotted from the read normalized bigwig files by the R package karyoploteR^124^ (version 1.16.0).

#### Replicate reproducibility and genome-wide binding correlation

To calculate correlation between replicates, we took the peaks called for individual replicates and used the db.count method from the R/BioConductor ChIPQC package^125^ (version 1.26.0) to count the number of sequencing reads in peaks with the following arguments: minimum mapping quality score of 30 (mapQCth=30), fragment size of 200 (fragmentSize=200), each peak must be present in both replicates (minOverlap=2), and report raw read count in the peaks (score= DBA_SCORE_READS). Pearson correlations were calculated, and scatter plots were made from log10(raw read counts +1) values from the two replicates.

To calculate pairwise correlation among all the DAP-seq and dDAP-seq samples, we first used the db.count method to combine the merged replicate GEM peaks reported for all samples to create a consensus peak set on which the sequencing reads were counted for each replicate, with the following arguments: minimum mapping quality score of 30 (mapQCth=30), fragment size of 200 (fragmentSize=200), each peak must be present in at least two samples (minOverlap=2), center the peaks and expand up- and downstream from the summit by 100 bp (summits=100), normalized to full library size (score=DBA_SCORE_NORMALIZED). From the consensus peak set, the regions that overlapped with the blacklist regions were removed and the regions that overlapped with the top 3000 most enriched peaks from each replicate were kept, resulting in a filtered consensus peak set. The normalized read counts at this filtered consensus peak set were extracted for each replicate, log2 transformed, and averaged between replicates. This created a log2 normalized read count vector for each sample. Pearson correlation was calculated between all pairs of samples to create the pairwise Pearson correlation matrix. With the ComplexHeatmap package^126^ (version 2.9.4), the Pearson correlation matrix was drawn as a heatmap with hierarchical clustering dendrogram calculated using the (1-Pearson correlation) values as distances between rows and columns and the average linkage method.

#### Motif discovery and scanning

For motif discovery of the most enriched 1000 peak sequences, the merged replicate GPS peaks were first filtered to remove peaks that overlapped with the blacklist regions. The filtered peaks were first ranked by the q-value of peak enrichment then by the fold enrichment values reported by GPS. DNA sequences were extracted from the TAIR10 reference genome for the 1000 highest ranked peaks. MEME-CHIP^81^ (version 5.3.0) was run on these peak sequences using the following parameters: “-meme-mod anr -meme-searchsize 0 -meme-nmotifs 5″. The top two PWM motifs reported by MEME (part of MEME-CHIP) were imported into R by the universalmotif package, aligned and extended by functions in the DiffLogos package^127^, and plotted by the ggseqlogo package^128^.

The GEM peak calling process included a motif discovery step that reported the enriched motifs found as KSM motif models. The KSM models from each DAP-seq or dDAP-seq samples were scanned for matches in the TAIR10 genome sequence using the KMAC tool that was part of the GEM package^90^.

Mapping peaks to target genes, GO enrichment and association with known targets: To predict the target genes using DAP- or dDAP-seq peaks, merged replicate GEM peaks for each sample or muMerge peaks for the bZIP groups were first filtered to remove peaks that overlapped with the blacklist regions. Using the “ClosestGene” method in the TFTargetCaller package^61^, we calculated the target scores and *q*-values for all the protein coding genes annotated in Araport11. Target *q*-values were used when comparing between samples or bZIP groups, while gene scores were used to rank genes for enrichment of GO terms or association with known target gene sets.

For GO enrichment analysis, we took the 2000 genes with the highest score for each sample, and used the clusterProfiler package^129^ to identify the top 5 most enriched GO categories in the Biological Process ontology annotated in the org.At.tair.db database and calculated the enrichment *P*-values of the GO terms in all the samples. The enrichment *P*-values were corrected by the Benjamini & Hochberg method and a matrix of −log10 adjusted *P*-values were created with enriched GO terms on the rows and DAP-seq or dDAP-seq samples on the columns and plotted as a heatmap by the ComplexHeatmap package^126^. Clustering dendrogram was obtained by hierarchical clustering of the column-centered and scaled −log10 *P*-value matrix with (1 – Pearson correlation) values between rows and columns as distances and the complete linkage method.

RNA-seq datasets for *bzipS1* mutant and for submergence treatment were downloaded from NCBI SRA. Quantification was performed by kallisto^130^ (version 0.45) and differential expression was determined by comparison to the appropriate wild type or mock treatment by sleuth^131^ (version 0.30). Dominant Patterns of gene expression specific to seed subregions and stages were downloaded from the supplemental data of Belmonte et al.^80^. The XL-mHG test^132,133^, implemented in the mhg_test function in the R package mhg, was used calculate the significance of association between target gene scores of individual samples or bZIP groups and gene sets from RNA-seq (*bzipS1* mutant, submergence treatment) or microarrays (seed subregion- and stage-specific genes).

Comparison of DAP-seq/dDAP-seq to targets of bZIP1 and bZIPS1: The classes of bZIP1 targets were obtained from supplementary materials of Para et al. ^63^ Read signal at the 2 kb region surrounding the TSS of the target genes were extracted from the bigWig files and plotted in a heatmap by the EnrichedHeatmap package^134^ (version 1.21.2).

Analysis of differential binding between homodimers and heterodimers: MANorm2_utils^135^ (version 1.0.0) were first used to find the number of sequencing reads of all S1 DAP-seq and S1:C dDAP-seq samples contained in the GPS peak regions that occurred in at least one sample. MAnorm2 R package^135^ (version 1.2.0) was used on this count matrix to perform normalization and differential binding analysis. For each pair of one S1:C and one S1, the replicates of each experiment were normalized followed by normalization between the experiments. A mean-variance curve fit for the pair of experiments was obtained using the local fit method and all genomic intervals. Degrees of freedom were estimated from the fitted curve using only bound intervals, and differential test was done to compare the S1:C to S1 experiments using the fitted mean-variance curve and degrees of freedom. The peaks that had adjusted *P*-values < 0.05 were called differentially bound. Regions that were significantly more enriched in S1:C dDAP-seq than S1 DAP-seq were called heterodimer specific and those that were significantly less enriched were call homodimer-specific. For motif discovery of bZIP53 heterodimer-specific peaks, sequences from the 2000 differentially bound peaks that had the highest fold changes when comparing each bZIP53:bZIPC to bZIP53 were used as input for MEME-CHIP^81^. For finding k-mer matches to KSM motif instances, KSM motif matches obtained previously that overlapped with 150 bp regions centered at the mid-point of 2000 most differentially bound regions were checked for matches to the k-mer sequences in one of the motif categories, and multiple matches to the same k-mer sequence in one peak were counted as one. The significance of enrichment of the k-mer sequences in heterodimer-specific peaks was calculated by Fisher exact test function in R, comparing the number of peaks with a k-mer sequence in heterodimer-specific peaks *vs*. the number of peaks with the same k-mer sequence in homodimer-specific peaks with alterative set to “greater”. The significance of enrichment of the k-mer sequence in homodimer-specific peaks was calculated by the R function fisher.test, comparing the number of peaks with a k-mer sequence in homodimer specific peaks *vs*. the number of peaks with the same k-mer sequence in heterodimer-specific peaks with alterative set to “greater”. Log2 odds ratios and *P*-values were obtained from returned values of the fisher.test function and *P*-values were adjusted for multiple testing by the Benjamini and Hochberg method.

### Reporting summary

Further information on research design is available in the Nature Portfolio Reporting Summary linked to this article.

## Supplementary information

Supplementary Information

Description of Additional Supplementary Files

Supplementary Data 1

Supplementary Data 2

Supplementary Data 3

Supplementary Data 4

Supplementary Data 5

Supplementary Data 6

Reporting Summary

## Data Availability

Raw and processed DAP-seq and dDAP-seq sequencing data have been deposited in the NCBI Gene Expression Omnibus (GEO) with accession number GSE198873. Source data are provided with this paper.

## Source data

Source Data

## References

[1] Inukai S, Kock KH, Bulyk ML (2017). Transcription factor–DNA binding: beyond binding site motifs. Curr. Opin. Genet. Dev..

[2] Colinas M, Goossens A (2018). Combinatorial transcriptional control of plant specialized metabolism. Trends Plant Sci..

[3] Boer DR (2014). Structural basis for DNA binding specificity by the auxin-dependent ARF transcription factors. Cell.

[4] Alerasool N, Leng H, Lin Z-Y, Gingras A-C, Taipale M (2022). Identification and functional characterization of transcriptional activators in human cells. Mol. Cell.

[5] Levo M, Segal E (2014). In pursuit of design principles of regulatory sequences. Nat. Rev. Genet..

[6] Strader L, Weijers D, Wagner D (2022). Plant transcription factors — being in the right place with the right company. Curr. Opin. Plant Biol..

[7] Shen N (2018). Divergence in DNA specificity among paralogous transcription factors contributes to their differential in vivo binding. Cell Syst..

[8] Shively Christian A, Liu J, Chen X, Loell K, Mitra Robi D (2019). Homotypic cooperativity and collective binding are determinants of bHLH specificity and function. Proc. Natl Acad. Sci. USA.

[9] De Val S (2008). Combinatorial regulation of endothelial gene expression by ets and forkhead transcription factors. Cell.

[10] Jolma A (2015). DNA-dependent formation of transcription factor pairs alters their binding specificity. Nature.

[11] Rodríguez-Martínez JA, Reinke AW, Bhimsaria D, Keating AE, Ansari AZ (2017). Combinatorial bZIP dimers display complex DNA-binding specificity landscapes. eLife.

[12] Kribelbauer JF, Rastogi C, Bussemaker HJ, Mann RS (2019). Low-affinity binding sites and the transcription factor specificity paradox in eukaryotes. Annu. Rev. Cell Dev. Biol..

[13] Jones S (2004). An overview of the basic helix-loop-helix proteins. Genome Biol..

[14] Lau OS (2018). Direct control of SPEECHLESS by PIF4 in the high-temperature response of stomatal development. Curr. Biol..

[15] Bai M-Y, Fan M, Oh E, Wang Z-Y (2012). A triple helix-loop-helix/basic helix-loop-helix cascade controls cell elongation downstream of multiple hormonal and environmental signaling pathways in Arabidopsis. Plant Cell.

[16] Amoutzias GD (2007). One billion years of bZIP transcription factor evolution: conservation and change in dimerization and DNA-binding site specificity. Mol. Biol. Evol..

[17] Lamb P, McKnight SL (1991). Diversity and specificity in transcriptional regulation: the benefits of heterotypic dimerization. Trends Biochem. Sci..

[18] Landschulz William H, Johnson Peter F, McKnight Steven L (1988). The leucine zipper: a hypothetical structure common to a new class of DNA binding proteins. Science.

[19] Riechmann JL (2000). Arabidopsis transcription factors: genome-wide comparative analysis among eukaryotes. Science.

[20] Dröge-Laser W, Snoek BL, Snel B, Weiste C (2018). The Arabidopsis bZIP transcription factor family—an update. Curr. Opin. Plant Biol..

[21] Mechta-Grigoriou F, Gerald D, Yaniv M (2001). The mammalian Jun proteins: redundancy and specificity. Oncogene.

[22] Hsu JC, Bravo R, Taub R (1992). Interactions among LRF-1, JunB, c-Jun, and c-Fos define a regulatory program in the G1 phase of liver regeneration. Mol. Cell. Biol..

[23] Foster R, Izawa T, Chua N-H (1994). Plant bZIP proteins gather at ACGT elements. FASEB J..

[24] Izawa T, Foster R, Chua N-H (1993). Plant bZIP protein DNA binding specificity. J. Mol. Biol..

[25] Martínez-García JF, Moyano E, Alcocer MJC, Martin C (1998). Two bZIP proteins from Antirrhinum flowers preferentially bind a hybrid C-box/G-box motif and help to define a new sub-family of bZIP transcription factors. Plant J..

[26] Williams ME, Foster R, Chua NH (1992). Sequences flanking the hexameric G-box core CACGTG affect the specificity of protein binding. Plant Cell.

[27] Satoh R, Fujita Y, Nakashima K, Shinozaki K, Yamaguchi-Shinozaki K (2004). A novel subgroup of bZIP proteins functions as transcriptional activators in hypoosmolarity-responsive expression of the ProDH gene in arabidopsis. Plant Cell Physiol..

[28] Satoh R, Nakashima K, Seki M, Shinozaki K, Yamaguchi-Shinozaki K (2002). ACTCAT, a novel cis-acting element for proline- and hypoosmolarity-responsive expression of the ProDH gene encoding proline dehydrogenase in Arabidopsis. Plant Physiol..

[29] Kim JS (2017). Genome-wide identification of grain filling genes regulated by the OsSMF1 transcription factor in rice. Rice.

[30] Onodera, Y. et al. A rice functional transcriptional activator, RISBZ1, responsible for endosperm-specific expression of storage protein genes through GCN4 motif. *J. Biol. Chem.*10.1074/jbc.M007405200 (2001). 10.1074/jbc.M007405200 11133985

[31] Ehlert A (2006). Two-hybrid protein–protein interaction analysis in Arabidopsis protoplasts: establishment of a heterodimerization map of group C and group S bZIP transcription factors. Plant J..

[32] Llorca CM (2015). The elucidation of the interactome of 16 Arabidopsis bZIP factors reveals three independent functional networks. PLoS ONE.

[33] Alonso R (2009). A pivotal role of the basic leucine zipper transcription factor bZIP53 in the regulation of Arabidopsis seed maturation gene expression based on heterodimerization and protein complex formation. Plant Cell.

[34] Kirchler T (2010). The role of phosphorylatable serine residues in the DNA-binding domain of Arabidopsis bZIP transcription factors. Eur. J. Cell Biol..

[35] Dietrich K (2011). Heterodimers of the Arabidopsis transcription factors bZIP1 and bZIP53 reprogram amino acid metabolism during low energy stress. Plant Cell.

[36] Baena-González E, Rolland F, Thevelein JM, Sheen J (2007). A central integrator of transcription networks in plant stress and energy signalling. Nature.

[37] Pedrotti L (2018). Snf1-RELATED KINASE1-controlled C/S1-bZIP signaling activates alternative mitochondrial metabolic pathways to ensure plant survival in extended darkness. Plant Cell.

[38] Weltmeier F (2006). Combinatorial control of Arabidopsis proline dehydrogenase transcription by specific heterodimerisation of bZIP transcription factors. EMBO J..

[39] Monteoliva MI, Rizzi YS, Cecchini NM, Hajirezaei M-R, Alvarez ME (2014). Context of action of proline dehydrogenase (ProDH) in the hypersensitive response of Arabidopsis. BMC Plant Biol..

[40] Barley BLZ (1998). a bZIP transcriptional activator that interacts with endosperm-specific gene promoters. Plant J..

[41] Oñate L (1999). a Seed-specific bZIP protein that interacts with BLZ1 in vivo and activates transcription from the GCN4-like motif of B-hordein promoters in barley endosperm*. J. Biol. Chem..

[42] Ellenberger TE, Brandl CJ, Struhl K, Harrison SC (1992). The GCN4 basic region leucine zipper binds DNA as a dimer of uninterrupted α Helices: Crystal structure of the protein-DNA complex. Cell.

[43] Chan IS, Fedorova AV, Shin JA (2007). The GCN4 bZIP targets noncognate gene regulatory sequences: quantitative investigation of binding at full and half sites. Biochemistry.

[44] Coey CT, Clark DJ (2022). A systematic genome-wide account of binding sites for the model transcription factor Gcn4. Genome Res..

[45] Lam HM, Peng SS, Coruzzi GM (1994). Metabolic regulation of the gene encoding glutamine-dependent asparagine synthetase in Arabidopsis thaliana. Plant Physiol..

[46] Weltmeier F (2009). Expression patterns within the Arabidopsis C/S1 bZIP transcription factor network: availability of heterodimerization partners controls gene expression during stress response and development. Plant Mol. Biol..

[47] O’Malley RC (2016). Cistrome and epicistrome features shape the regulatory DNA landscape. Cell.

[48] Bartlett A (2017). Mapping genome-wide transcription-factor binding sites using DAP-seq. Nat. Protoc..

[49] Jakoby M (2002). bZIP transcription factors in *Arabidopsis*. Trends Plant Sci..

[50] Deppmann CD (2004). Dimerization specificity of all 67 B-ZIP motifs in Arabidopsis thaliana: a comparison to Homo sapiens B-ZIP motifs. Nucleic Acids Res..

[51] Fassler J (2002). B-ZIP proteins encoded by the drosophila genome: evaluation of potential dimerization partners. Genome Res..

[52] Berger MF, Bulyk ML (2009). Universal protein-binding microarrays for the comprehensive characterization of the DNA-binding specificities of transcription factors. Nat. Protoc..

[53] Park PJ (2009). ChIP–seq: advantages and challenges of a maturing technology. Nat. Rev. Genet..

[54] Worsley Hunt R, Wasserman WW (2014). Non-targeted transcription factors motifs are a systemic component of ChIP-seq datasets. Genome Biol..

[55] Lara P (2003). Synergistic activation of seed storage protein gene expression in Arabidopsis by ABI3 and two bZIPs related to OPAQUE2*. J. Biol. Chem..

[56] Kaminaka H (2006). bZIP10-LSD1 antagonism modulates basal defense and cell death in Arabidopsis following infection. EMBO J..

[57] Kang SG, Price J, Lin P-C, Hong JC, Jang J-C (2010). The Arabidopsis bZIP1 transcription factor is involved in sugar signaling, protein networking, and DNA binding. Mol. Plant.

[58] Jain P (2017). A-ZIP53, a dominant negative reveals the molecular mechanism of heterodimerization between bZIP53, bZIP10 and bZIP25 involved in Arabidopsis seed maturation. Sci. Rep..

[59] Mergner J (2020). Mass-spectrometry-based draft of the Arabidopsis proteome. Nature.

[60] Mair A (2015). SnRK1-triggered switch of bZIP63 dimerization mediates the low-energy response in plants. eLife.

[61] Sikora-Wohlfeld W, Ackermann M, Christodoulou EG, Singaravelu K, Beyer A (2013). Assessing computational methods for transcription factor target gene identification based on ChIP-seq data. PLOS Comput. Biol..

[62] Wagner, F. The XL-mHG test for gene set enrichment. *PeerJ Preprints* **5**, e1962v3 (2017).

[63] Para A (2014). Hit-and-run transcriptional control by bZIP1 mediates rapid nutrient signaling in *Arabidopsis*. Proc. Natl Acad. Sci. USA.

[64] Dröge-Laser W, Weiste C (2018). The C/S1 bZIP network: a regulatory hub orchestrating plant energy homeostasis. Trends Plant Sci..

[65] Borisjuk L, Rolletschek H (2009). The oxygen status of the developing seed. N Phytol..

[66] Choi BY (2022). The Chlamydomonas bZIP transcription factor BLZ8 confers oxidative stress tolerance by inducing the carbon-concentrating mechanism. Plant Cell.

[67] Drew MC (1997). Oxygen deficiency and root metabolism: injury and acclimation under hypoxia and anoxia. Annu. Rev. Plant Physiol. Plant Mol. Biol..

[68] Geigenberger P (2003). Response of plant metabolism to too little oxygen. Curr. Opin. Plant Biol..

[69] Meng X (2020). Mitochondrial signalling is critical for acclimation and adaptation to flooding in Arabidopsis thaliana. Plant J..

[70] Weiste C, Dröge-Laser W (2014). The Arabidopsis transcription factor bZIP11 activates auxin-mediated transcription by recruiting the histone acetylation machinery. Nat. Commun..

[71] Cutler SR, Rodriguez PL, Finkelstein RR, Abrams SR (2010). Abscisic acid: emergence of a core signaling network. Annu. Rev. Plant Biol..

[72] Finkelstein RR, Gampala SSL, Rock CD (2002). Abscisic acid signaling in seeds and seedlings. Plant Cell.

[73] Song L (2016). A transcription factor hierarchy defines an environmental stress response network. Science.

[74] Goda H (2008). The AtGenExpress hormone and chemical treatment data set: experimental design, data evaluation, model data analysis and data access. Plant J..

[75] Yamaguchi-Shinozaki K, Shinozaki K (1994). A novel cis-acting element in an Arabidopsis gene is involved in responsiveness to drought, low-temperature, or high-salt stress. Plant Cell.

[76] Msanne J, Lin J, Stone JM, Awada T (2011). Characterization of abiotic stress-responsive Arabidopsis thaliana RD29A and RD29B genes and evaluation of transgenes. Planta.

[77] Tiwari SB, Hagen G, Guilfoyle T (2003). The roles of auxin response factor domains in auxin-responsive transcription. Plant Cell.

[78] Yoo S-D, Cho Y-H, Sheen J (2007). Arabidopsis mesophyll protoplasts: a versatile cell system for transient gene expression analysis. Nat. Protoc..

[79] Zhang, L. et al. A novel wheat C-bZIP gene, TabZIP14-B, participates in salt and freezing tolerance in transgenic plants. *Front. Plant Sci.* **8**, 710 (2017). 10.3389/fpls.2017.00710 PMC5422549 28536588

[80] Belmonte Mark F (2013). Comprehensive developmental profiles of gene activity in regions and subregions of the Arabidopsis seed. Proc. Natl Acad. Sci. USA.

[81] Machanick P, Bailey TL (2011). MEME-ChIP: motif analysis of large DNA datasets. Bioinformatics.

[82] Després C, DeLong C, Glaze S, Liu E, Fobert PR (2000). The Arabidopsis NPR1/NIM1 protein enhances the DNA binding activity of a subgroup of the TGA family of bZIP transcription factors. Plant Cell.

[83] Zhang Y, Fan W, Kinkema M, Li X, Dong X (1999). Interaction of NPR1 with basic leucine zipper protein transcription factors that bind sequences required for salicylic acid induction of the PR-1 gene. Proc. Natl Acad. Sci. USA.

[84] Sullivan AlessandraM (2014). Mapping and dynamics of regulatory DNA and transcription factor networks in A. thaliana. Cell Rep..

[85] Weirauch MatthewT (2014). Determination and inference of eukaryotic transcription factor sequence specificity. Cell.

[86] Franco-Zorrilla JM (2014). DNA-binding specificities of plant transcription factors and their potential to define target genes. Proc. Natl Acad. Sci. USA.

[87] Mathelier A, Wasserman WW (2013). The next generation of transcription factor binding site prediction. PLoS Comput. Biol..

[88] Keilwagen J, Grau J (2015). Varying levels of complexity in transcription factor binding motifs. Nucleic Acids Res..

[89] Ruan S, Stormo GD (2018). Comparison of discriminative motif optimization using matrix and DNA shape-based models. BMC Bioinform..

[90] Guo, Y., Tian, K., Zeng, H., Guo, X. & Gifford, D. K. A novel k-mer set memory (KSM) motif representation improves regulatory variant prediction. *Genome Res.* **28**, 891–900 (2018). 10.1101/gr.226852.117 PMC5991515 29654070

[91] Isakova A (2017). SMiLE-seq identifies binding motifs of single and dimeric transcription factors. Nat. Methods.

[92] Bartlett ME (2017). Changing MADS-box transcription factor protein–protein interactions as a mechanism for generating floral morphological diversity. Integr. Comp. Biol..

[93] Hugouvieux V, Zubieta C (2018). MADS transcription factors cooperate: complexities of complex formation. J. Exp. Bot..

[94] Lai X (2020). Genome-wide binding of SEPALLATA3 and AGAMOUS complexes determined by sequential DNA-affinity purification sequencing. Nucleic Acids Res..

[95] Ptashne M, Gann A (1997). Transcriptional activation by recruitment. Nature.

[96] Hope Ia Fau - Struhl, K. & Struhl, K. Functional dissection of a eukaryotic transcriptional activator protein, GCN4 of yeast. *Cell* **46**, 885-94 (1986). 10.1016/0092-8674(86)90070-x 3530496

[97] Brent R Fau - Ptashne, M. & Ptashne, M. A eukaryotic transcriptional activator bearing the DNA specificity of a prokaryotic repressor. *Cell*10.1016/0092-8674(86)90070-x (1985). 10.1016/0092-8674(85)90246-6 3907859

[98] Walther D, Brunnemann R, Selbig J (2007). The regulatory code for transcriptional response diversity and its relation to genome structural properties in A. thaliana. PLoS Genet..

[99] Llorca, C. M., Potschin, M. & Zentgraf, U. bZIPs and WRKYs: two large transcription factor families executing two different functional strategies. *Front. Plant Sci.* **5**, 169 (2014). 10.3389/fpls.2014.00169 PMC4012195 24817872

[100] Corrêa LGG (2008). The role of bZIP transcription factors in green plant evolution: adaptive features emerging from four founder genes. PLoS ONE.

[101] Peviani A, Lastdrager J, Hanson J, Snel B (2016). The phylogeny of C/S1 bZIP transcription factors reveals a shared algal ancestry and the pre-angiosperm translational regulation of S1 transcripts. Sci. Rep..

[102] Bowles AMC, Bechtold U, Paps J (2020). The origin of land plants is rooted in two bursts of genomic novelty. Curr. Biol..

[103] Hollenbeck JJ, Oakley MG (2000). GCN4 binds with high affinity to DNA sequences containing a single consensus half-site. Biochemistry.

[104] Zhu C (2009). High-resolution DNA-binding specificity analysis of yeast transcription factors. Genome Res..

[105] Gordân R (2011). Curated collection of yeast transcription factor DNA binding specificity data reveals novel structural and gene regulatory insights. Genome Biol..

[106] Coey, C. T. & Clark, D. J. A systematic genome-wide account of binding sites for the model transcription factor Gcn4. *Genome Res.* **32**, 367–377 (2021). 10.1101/gr.276080.121 PMC8805717 34916251

[107] Chow C-N (2019). PlantPAN3.0: a new and updated resource for reconstructing transcriptional regulatory networks from ChIP-seq experiments in plants. Nucleic Acids Res..

[108] Kim SY, Wu R (1990). Multiple protein factors bind to a rice glutelin promoter region. Nucleic Acids Res..

[109] López García de Lomana A (2015). Transcriptional program for nitrogen starvation-induced lipid accumulation in Chlamydomonas reinhardtii. Biotechnol. Biofuels.

[110] Li C (2015). Genome-wide characterization of cis-acting dna targets reveals the transcriptional regulatory framework of opaque2 in maize. Plant Cell.

[111] Hurst H (1994). Transcription factors: I. bZIP proteins. Protein Profiles.

[112] Ezer D (2017). The G-box transcriptional regulatory code in Arabidopsis. Plant Physiol..

[113] Sielemann J, Wulf D, Schmidt R, Brautigam A (2021). Local DNA shape is a general principle of transcription factor binding specificity in Arabidopsis thaliana. Nat. Commun..

[114] Keefe AD, Wilson DS, Seelig B, Szostak JW (2001). One-step purification of recombinant proteins using a nanomolar-affinity streptavidin-binding peptide, the SBP-tag. Protein Expr. Purif..

[115] Glenn TC (2019). Adapterama I: universal stubs and primers for 384 unique dual-indexed or 147,456 combinatorially-indexed Illumina libraries (iTru & iNext). PeerJ.

[116] Hartig, F. DHARMa: residual diagnostics for hierarchical (multi-level/mixed) regression models. https://cran.r-project.org/web/packages/DHARMa/index.html (2022).

[117] Lenth, R. et al. EMMEANS: estimated marginal means, aka least-squares means. https://cran.r-project.org/web/packages/emmeans/index.html (2022).

[118] Martin, M. Cutadapt removes adapter sequences from high-throughput sequencing reads. *EMBnet.journal*; *Vol 17, No 1: Next Generation Sequencing Data AnalysisDO − 10.14806/ej.17.1.200* (2011).

[119] Langmead B, Salzberg SL (2012). Fast gapped-read alignment with Bowtie 2. Nat. Methods.

[120] Guo Y, Mahony S, Gifford DK (2012). High resolution genome wide binding event finding and motif discovery reveals transcription factor spatial binding constraints. PLoS Comput. Biol..

[121] Zhang Y (2008). Model-based analysis of ChIP-Seq (MACS). Genome Biol..

[122] Rubin JD (2021). Transcription factor enrichment analysis (TFEA) quantifies the activity of multiple transcription factors from a single experiment. Commun. Biol..

[123] Ramírez F (2016). deepTools2: a next generation web server for deep-sequencing data analysis. Nucleic Acids Res..

[124] Gel B, Serra E (2017). karyoploteR: an R/Bioconductor package to plot customizable genomes displaying arbitrary data. Bioinformatics.

[125] Carroll, T. S., Liang, Z., Salama, R., Stark, R. & de Santiago, I. Impact of artifact removal on ChIP quality metrics in ChIP-seq and ChIP-exo data. *Front. Genet.* **5**, 75 (2014). 10.3389/fgene.2014.00075 PMC3989762 24782889

[126] Gu Z, Eils R, Schlesner M (2016). Complex heatmaps reveal patterns and correlations in multidimensional genomic data. Bioinformatics.

[127] Nettling M (2015). DiffLogo: a comparative visualization of sequence motifs. BMC Bioinform..

[128] Wagih O (2017). ggseqlogo: a versatile R package for drawing sequence logos. Bioinformatics.

[129] Wu T (2021). clusterProfiler 4.0: a universal enrichment tool for interpreting omics data. Innovation.

[130] Bray NL, Pimentel H, Melsted P, Pachter L (2016). Near-optimal probabilistic RNA-seq quantification. Nat. Biotechnol..

[131] Pimentel H, Bray NL, Puente S, Melsted P, Pachter L (2017). Differential analysis of RNA-seq incorporating quantification uncertainty. Nat. Methods.

[132] Eden E, Lipson D, Yogev S, Yakhini Z (2007). Discovering motifs in ranked lists of DNA sequences. PLoS Comput. Biol..

[133] Wagner F (2015). GO-PCA: an unsupervised method to explore gene expression data using prior knowledge. PLoS ONE.

[134] Gu Z, Eils R, Schlesner M, Ishaque N (2018). EnrichedHeatmap: an R/Bioconductor package for comprehensive visualization of genomic signal associations. BMC Genomics.

[135] Tu S (2021). MAnorm2 for quantitatively comparing groups of ChIP-seq samples. Genome Res..

