# Supplementary material for: Double DAP-seq uncovered synergistic DNA binding of interacting bZIP transcription factors
Source: Nat Commun. 2023 May 5;14:2600. doi: 10.1038/s41467-023-38096-2 (PMC10163045; doi:10.1038/s41467-023-38096-2)
Supplement: Supplementary file 1 — Supplementary Information [file 41467_2023_38096_MOESM1_ESM.pdf]

Supplementary information

Double DAP-seq uncovered synergistic DNA binding of interacting bZIP transcription factors

Miaomiao Li[1], Tao Yao[2], Wanru Lin[1], Will E. Hinckley[1], Mary Galli[3], Wellington Muchero[2], Andrea Gallavotti[3], Jin-Gui Chen[2] and Shao-shan Carol Huang[1,*]

[1] Center for Genomics and Systems Biology, Department of Biology, New York University, New York, NY 10003, USA

[2] Biosciences Division, Oak Ridge National Laboratory, Oak Ridge, TN 37831, USA

[3] Waksman Institute of Microbiology, Rutgers University, Piscataway, NJ 08854-8020, USA.

*Correspondence: s.c.huang@nyu.edu

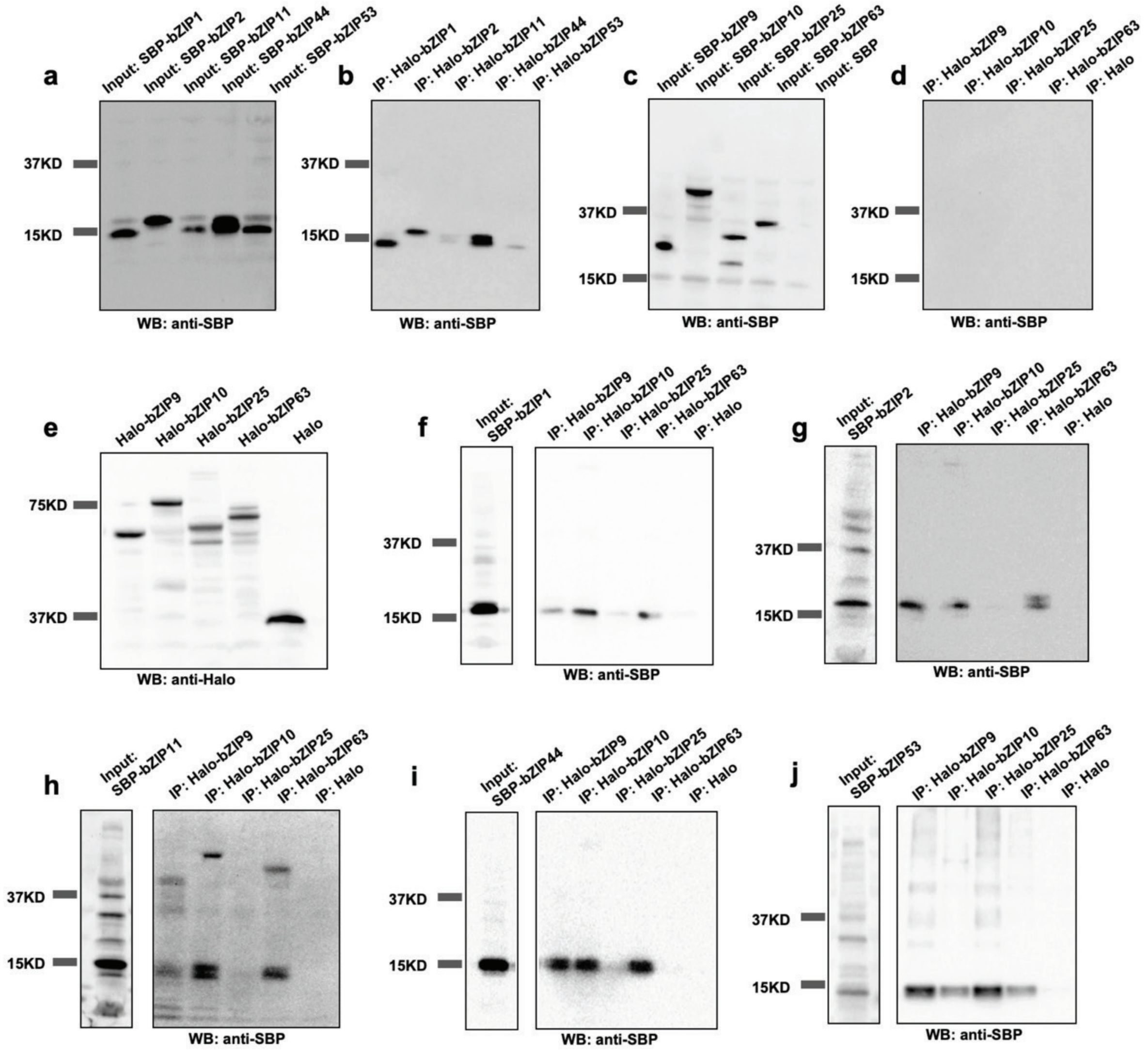

Supplementary Figure 1: C/S1 bZIPs form homodimers and heterodimers. a and **b** S1 bZIP fused to SBPTag (SBP-bZIPS1) physically interact with bZIPS1 fused to HaloTag (Halo-bZIPS1) in *in vitro* pull-down assays. SBP-bZIPS1 was mixed with Halo-bZIPS1 (input samples in a) and affinity purification was carried out by HaloTag ligand-coupled magnetic beads (b). SBP-bZIPS1 was detected by an anti-SBP antibody. **c** and **d** SBP-bZIPC cannot interact with Halo-bZIPC in *in vitro* pull-down assays. SBP-bZIPC was mixed with Halo-bZIPC (input samples in c) and affinity purification was performed by HaloTag ligand-coupled magnetic beads (d). SBP-bZIPC was detected by an anti-SBP antibody. **e** Expression of Halo-bZIPC proteins and HaloTag was detected by an anti-Halo antibody. **f-j** SBP-bZIPS1 physically interacts with Halo-bZIPC *in vitro* pull-down assays. SBP-bZIPS1 was mixed with Halo-bZIPC (lanes labeled input) and affinity purification was performed by HaloTag ligand-coupled magnetic beads (lanes

labeled IP). SBP-bZIPS1 was detected by an anti-SBP antibody. The HaloTag empty vector was used as a negative control. Pull-down assays were done side-by-side with the dDAP-seq experiments and repeated more than two times. WB: western blotting. IP: immunoprecipitation.

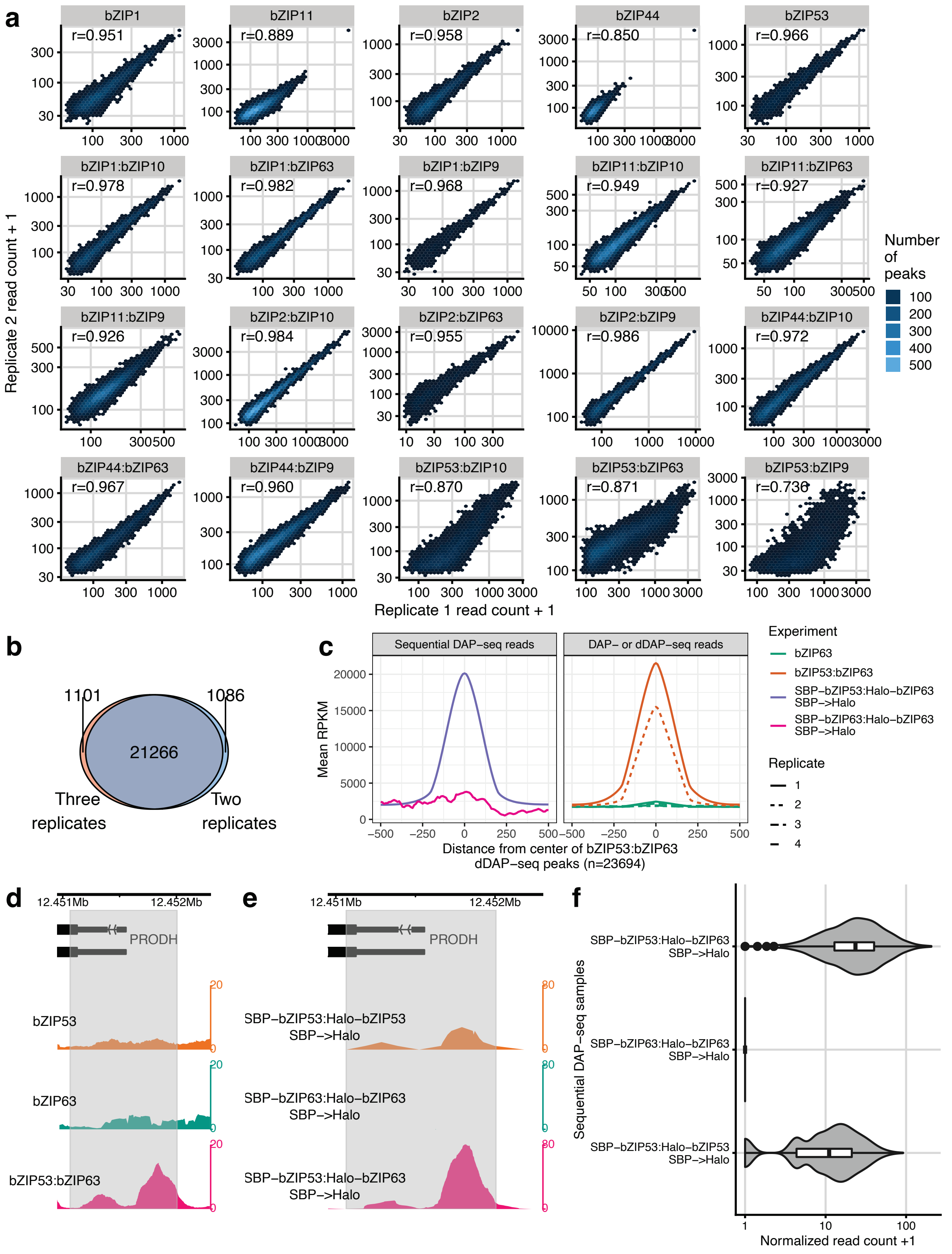

Supplementary Figure 2: Evaluation of dDAP-seq method for identifying S1:C binding

sites. a Correlation between replicates across all the S1 DAP-seq and S1:C dDAP-seq samples. The number of reads in peaks in replicate 1 (x-axis) and the number of reads in the same peaks in replicate 2 (y-axis) for the two replicates of each sample are plotted as hexagonal heatmaps of 2d bin counts. Pearson correlation values are indicated for each sample. **b** Venn diagram of peaks identified for bZIP53:bZIP63 using two replicates vs. three replicates. **c** Normalized read counts from sequential DAP-seq, dDAP-seq and DAP-seq of bZIP53 and bZIP63 at 500 bp +/- of centers of peaks identified from bZIP53:bZIP63 dDAP-seq. **d** DAP-, dDAP- seq binding signal of bZIP53, bZIP63, and bZIP53:bZIP63 at the promoter of *PRODH*. **e** Binding signal of sequential DAP-seq of bZIP53 and bZIP63 pairs at the promoter of *PRODH*. Gray rectangle marks a region previously characterized in reporter assays (Veerabagu et al., Mol Plant, 2014). **f** Comparing bZIP53:bZIP63 dDAP-seq to bZIP53 DAP-seq identified bZIP53:bZIP63 heterodimer-specific peaks (see Fig. 5) that also increase binding comparing bZIP53:bZIP63 sDAP-seq to bZIP53:bZIP53 sDAP-seq. x-axis is in log-scale. In the box-and-whisker plots, lower and upper hinges correspond to the first and third quantiles, lower and upper whiskers extend to at most 1.5 times the inter-quantile range, and data points beyond the end of whiskers are plotted individually. n=2,126 differentially bound peaks from comparing two replicates of bZIP53:bZIP63 dDAP-seq to two replicates of bZIP53 DAP-seq.

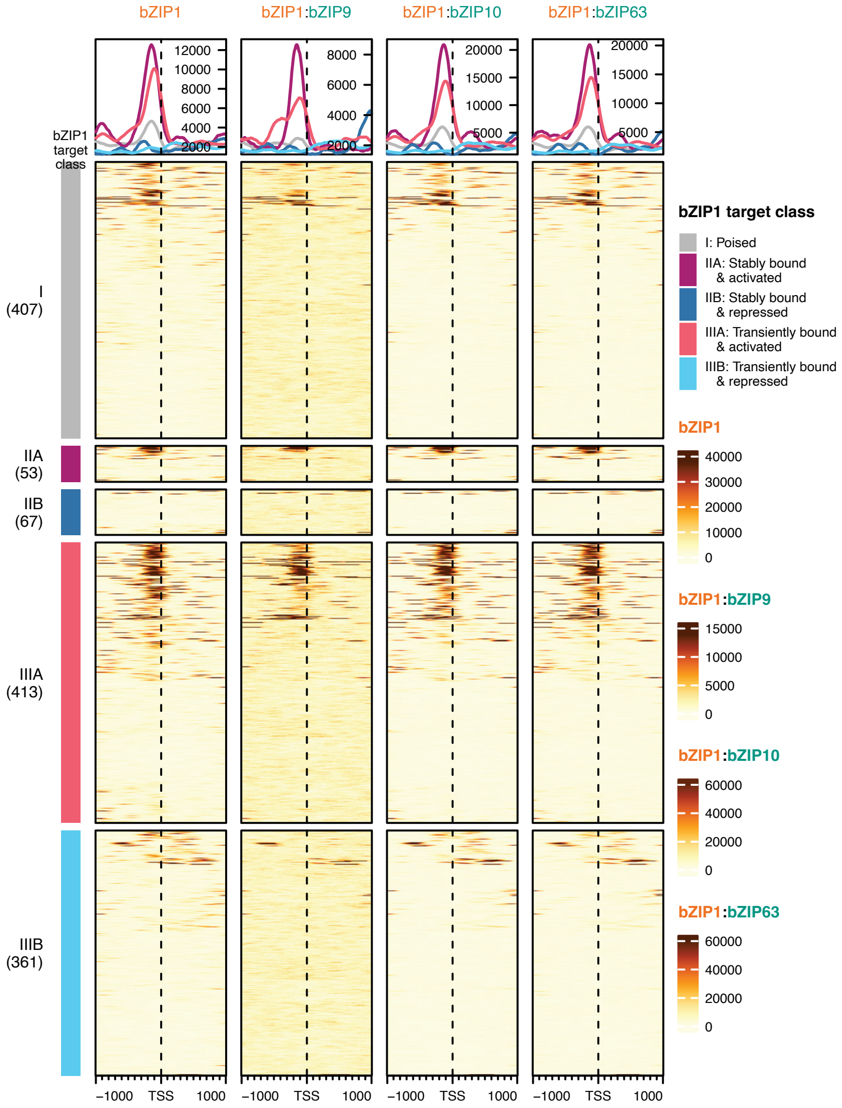

Supplementary Figure 3: Heatmap showing the DAP-seq and dDAP-seq binding signal of bZIP1 homodimer and heterodimers centered at the transcription start site (TSS) of bZIP1 targets. Left panel indicates the class of bZIP1 targets determined from ChIP-seq and time course TARGET experiments to be transiently or stably bound and activated or repressed by bZIP1.

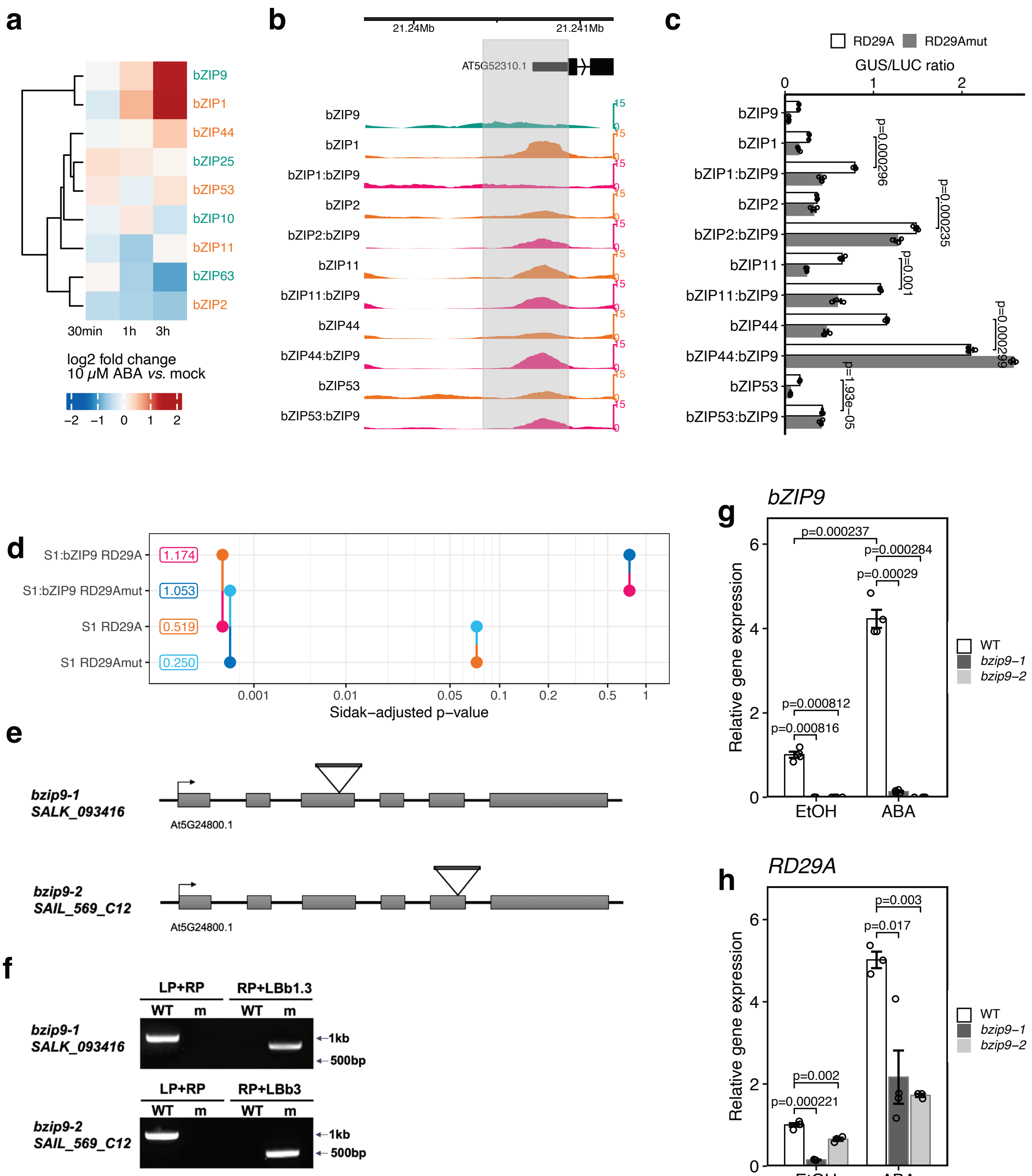

Supplemental Figure 4: dDAP-seq implicates bZIP9 functions in ABA response. a Changes in expression of C/S1 bZIPs in an ABA treatment time course of *Arabidopsis* seedlings. Fold changes were calculated by 10 μM ABA treatment for 30 min, 1h and 3h and relative to mock-treated control at the same time point. **b** Binding signal of bZIPS1:bZIP9 and bZIPS1 at the

promoter region of the ABA response marker genes *RD29A* (AT5G52300). Gray rectangle marks the region used in the reporter assay. **c** Transient expression of bZIP9 with each S1 bZIP resulted in statistically significant increased expression of the *RD29A::GUS* reporter compared to bZIPS1 alone. n=3 replicates. Data are normalized to luciferase control (LUC). Bar charts represent mean ± standard error (SE). P-values shown were computed by two-sided t-tests without adjustments for multiple comparisons. **d** Pairwise p-value plot comparing reporter activation in four combinations of effectors (S1 alone and S1 with bZIP9) and reporters (RD29A and RD2Amut; Supplementary Table 6). The number next to each combination is the estimated mean activation averaged over S1 bZIPs. Each line segment connects a pair of combinations being compared, with point and half-line segment drawn for one combination in the color of the other combination in the pair. The x-axis location of the line segment indicates the P-value of the comparison. P-values were computed by two-sided tests comparing the estimated marginal means of the indicated factor combinations followed by Sidak correction for multiple comparisons. **e** Characterization of *Arabidopsis bzip9* T-DNA insertion mutants. Schematic representation of two individual *bzip9* T-DNA insertion lines. Gray boxes represent exons and lines show introns and UTRs, arrows represent translation stat sites, and the triangles indicate the T-DNA insertion sites. **f** Genotyping of *bzip9-1* and *bzip9-2* mutants. Primers LP and RP amplified the wild-type gene fragments, and primers RP and LBb1.3 amplified the gene fragments from the mutant lines. **g** Gene expression analysis of wild-type (WT) and two *bzip9* mutant lines. qRT-PCR analysis of the transcript abundance of *bZIP9* in WT and two *bzip9* mutant lines in control or 50 μM ABA treatment. n=3 independent experiments. The bar chart represents mean ± SE. P-values shown were computed by two-sided t-tests without adjustments for multiple comparisons. **h** Expression of *RD29A* genes in wild-type (WT) and *bzip9* mutants after treatment of 50 μM ABA for 3 h with ethanol (EtOH) as negative control. Error bars indicate SE of three independent seedling pools. P-values shown were computed by two-sided t-tests without adjustments for multiple comparisons.

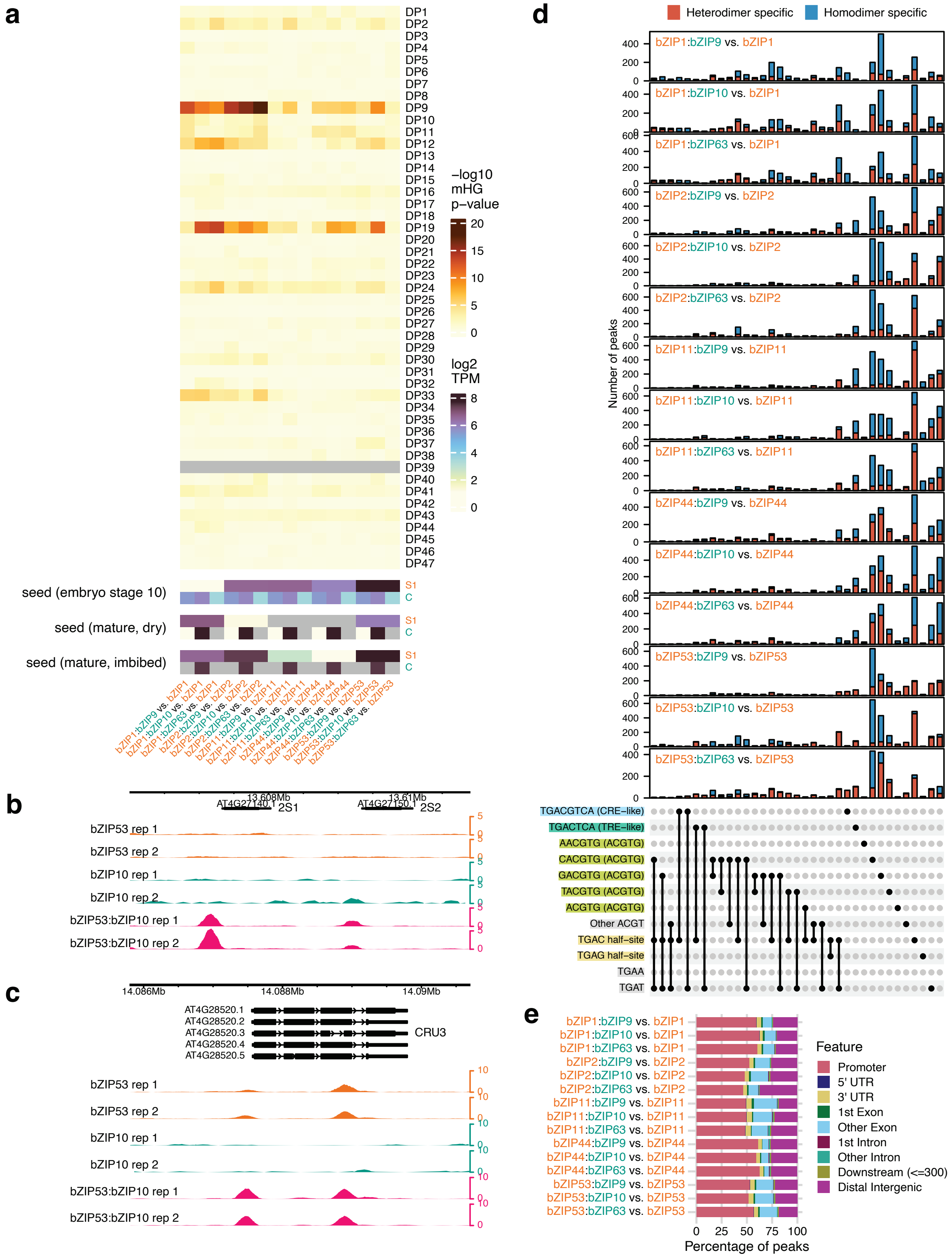

Supplementary Figure 5: Target genes and sequence specificities of C/S1 heterodimer-

specific binding sites. a Minimal hypergeometric (mHG) test P-values of testing association between S1:C heterodimer-specific target genes and gene sets that show distinct dominant patterns (DP) of gene expression in sub-regions at specific stages of the developing seed. P-values shown were computed by the mHG enrichment tests without adjustment for multiple comparisons. **b** and **c** bZIP53:bZIP10 heterodimer binding by dDAP-seq at the promoter regions of *2S1* and *2S2* (b) and *CRU3* (c). **d** KSM motif occurrences in peaks that showed increased binding comparing each S1:C to S1 (heterodimer-specific) or decreased binding (homodimer-specific). **e** Distribution of heterodimer-specific peaks relative to genome annotation features. Promoter regions were defined as ±1 kb from the TSS.

	Motif 1	Motif 2	Motif 3
**Yeast GCN4**<br>Zhu et al., 2009<br>Gordân et al., 2011<br>Coey and Clark 2021	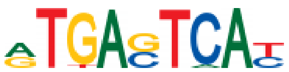<br>UniPROBE UP00285	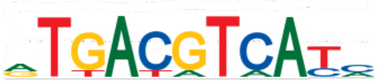<br>GCN4 secondary motif<br>Gordân et al., 2011 Table S6	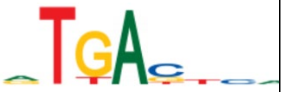<br>UniPROBE UP02285
**Algae bZIPs**<br>López García de Lomana et al. 2015	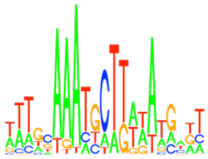<br>Chlamy-Portal Cre05.g238250 motif_0019_1	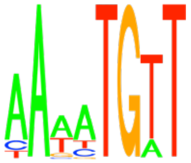<br>Chlamy-Portal Cre12.g510200 motif_0006_2	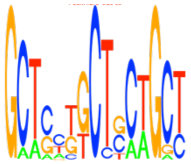<br>Chlamy-Portal Cre17.g746547 motif_0111_2
**Maize Group C ZmbZIP1/O2**<br>Li et al., 2015	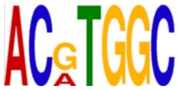<br>PCBase (PlantPAN 3.0) GSE63991 CSmatrixID_2200	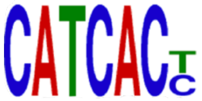<br>PCBase (PlantPAN 3.0) GSE63991 CSmatrixID_2201	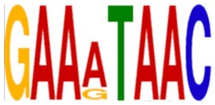<br>PCBase (PlantPAN 3.0) GSE63991 CSmatrixID_2202
**Rice Group C OsbZIP58/SMF1**<br>Kim et al., 2017	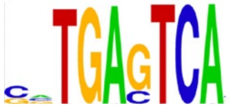<br>Kim et al., 2017	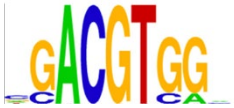<br>Kim et al., 2017	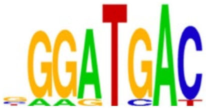<br>Kim et al., 2017
**Arabidopsis Group C/S1**	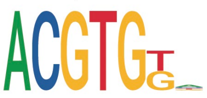<br>This paper	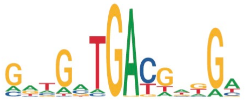<br>This paper	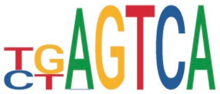<br>This paper

Supplemental Figure 6: bZIPs from yeast and multiple plant species bind to GCN4-like motifs and ACGT elements. Data sources are: protein binding microarray (PBM; Zhu et al., 2009 and Gordân et al., 2011) and G-SELEX (Coey and Clark 2021) for yeast collected in UniPROBE (Hume et al., 2015), regulatory network model of algae in Chlamy-Portal (López García de Lomana et al., 2015), ChIP-seq in maize (Li et al., 2015) collected in PCBase (Chow et al., 2019), PBM in rice (Kim et al., 2017) and DAP- and dDAP-seq in *Arabidopsis* (this paper).
