# Supplementary material for: Double DAP-seq uncovered synergistic DNA binding of interacting bZIP transcription factors
Source: Nat Commun. 2023 May 5;14:2600. doi: 10.1038/s41467-023-38096-2 (PMC10163045; doi:10.1038/s41467-023-38096-2)
Supplement: Supplementary file 2 — Description of Additional Supplementary Files [file 41467_2023_38096_MOESM2_ESM.pdf]

Description of Additional Supplementary Files:

Supplementary Data 1: DAP-seq, double DAP-seq and sequential DAP-seq samples for Arabidopsis C/S1 bZIP transcription factors

Supplementary Data 2: Differentially expressed genes (DEGs) in the bzipS1 mutant are grouped into six clusters by DAP/dDAP

Supplementary Data 3: Differential binding analysis comparing S1:C dDAP-seq and S1 DAP-seq and annotated genes

Supplementary Data 4: Number of occurrences of motifs in different categories in homodimer- and heterodimer-specific peaks

Supplementary Data 5: Primers used in this study

Supplementary Data 6: Sequences of the promoter regions used in reporter assay experiments
