# Supplementary material for: Double DAP-seq uncovered synergistic DNA binding of interacting bZIP transcription factors
Source: Nat Commun. 2023 May 5;14:2600. doi: 10.1038/s41467-023-38096-2 (PMC10163045; doi:10.1038/s41467-023-38096-2)
Supplement: Supplementary file 9 — Reporting Summary [file 41467_2023_38096_MOESM9_ESM.pdf]

nature portfolio

Corresponding author(s): Shao-shan Carol Huang

Last updated by author(s): April 6, 2023

Reporting Summary

Nature Portfolio wishes to improve the reproducibility of the work that we publish. This form provides structure for consistency and transparency in reporting. For further information on Nature Portfolio policies, see our Editorial Policies and the Editorial Policy Checklist.

Statistics

For all statistical analyses, confirm that the following items are present in the figure legend, table legend, main text, or Methods section.

n/a	Confirmed	
☐	☒	The exact sample size (*n*) for each experimental group/condition, given as a discrete number and unit of measurement
☐	☒	A statement on whether measurements were taken from distinct samples or whether the same sample was measured repeatedly
☐	☒	The statistical test(s) used AND whether they are one- or two-sided<br>*Only common tests should be described solely by name; describe more complex techniques in the Methods section.*
☐	☒	A description of all covariates tested
☐	☒	A description of any assumptions or corrections, such as tests of normality and adjustment for multiple comparisons
☐	☒	A full description of the statistical parameters including central tendency (e.g. means) or other basic estimates (e.g. regression coefficient) AND variation (e.g. standard deviation) or associated estimates of uncertainty (e.g. confidence intervals)
☐	☒	For null hypothesis testing, the test statistic (e.g. *F*, *t*, *r*) with confidence intervals, effect sizes, degrees of freedom and *P* value noted<br>*Give P values as exact values whenever suitable.*
☒	☐	For Bayesian analysis, information on the choice of priors and Markov chain Monte Carlo settings
☒	☐	For hierarchical and complex designs, identification of the appropriate level for tests and full reporting of outcomes
☒	☐	Estimates of effect sizes (e.g. Cohen's *d*, Pearson's *r*), indicating how they were calculated

Our web collection on statistics for biologists contains articles on many of the points above.

Software and code

Policy information about availability of computer code

Data collection	All software packages and versions used are described in the NCBI GEO record.
Data analysis	All software packages and versions used are described and cited in the Methods section.

For manuscripts utilizing custom algorithms or software that are central to the research but not yet described in published literature, software must be made available to editors and reviewers. We strongly encourage code deposition in a community repository (e.g. GitHub). See the Nature Portfolio guidelines for submitting code & software for further information.

Data

Policy information about availability of data

All manuscripts must include a data availability statement. This statement should provide the following information, where applicable:

- Accession codes, unique identifiers, or web links for publicly available datasets
- A description of any restrictions on data availability
- For clinical datasets or third party data, please ensure that the statement adheres to our policy

Raw and processed DAP-seq and dDAP-seq sequencing data have been deposited in the Gene Expression Omnibus (GEO) with accession number GSE198873.

Human research participants

Policy information about studies involving human research participants and Sex and Gender in Research.

Reporting on sex and gender	Not applicable.
Population characteristics	Not applicable.
Recruitment	Not applicable.
Ethics oversight	Not applicable.

Note that full information on the approval of the study protocol must also be provided in the manuscript.

Field-specific reporting

Please select the one below that is the best fit for your research. If you are not sure, read the appropriate sections before making your selection.

☒ Life sciences ☐ Behavioural & social sciences ☐ Ecological, evolutionary & environmental sciences

For a reference copy of the document with all sections, see nature.com/documents/nr-reporting-summary-flat.pdf

Life sciences study design

All studies must disclose on these points even when the disclosure is negative.

Sample size	Sample size was determined based on field convention and recommended measures of reproducibility.
Data exclusions	Data were excluded based on pre-determined metrics quality control measures.
Replication	Reproducibility was measured using metrics recommended by published guidelines for the data types.
Randomization	Not applicable.
Blinding	Not applicable.

Reporting for specific materials, systems and methods

We require information from authors about some types of materials, experimental systems and methods used in many studies. Here, indicate whether each material, system or method listed is relevant to your study. If you are not sure if a list item applies to your research, read the appropriate section before selecting a response.

Materials & experimental systems

n/a	Involved in the study	
☐	☒	Antibodies
☒	☐	Eukaryotic cell lines
☒	☐	Palaeontology and archaeology
☒	☐	Animals and other organisms
☒	☐	Clinical data
☒	☐	Dual use research of concern

Methods

n/a	Involved in the study	
☒	☐	ChIP-seq
☒	☐	Flow cytometry
☒	☐	MRI-based neuroimaging

Antibodies

Antibodies used	anti-HaloTag monoclonal antibody (Promega, G9211); anti-SBPTag mouse anti-human antibody (MilliporeSigma, MAB10764)
Validation	G9211:<br>Application: Immunological detection of intact HaloTag® fusion proteins, specifically for Western blotting purposes. Little to no cross-reactivity with other non-HaloTag® proteins. Western blot detection of EZH2-HaloTag® (HT) fusion protein (~120kDa) using AntiHaloTag® Monoclonal Antibody (mAb). Blot shows specific and robust detection of the HaloTag® fusion from HEK293T cell lysate.<br><br>MAB10764:

Specificity: Demonstrated to react with cells expressing the SBP tag. This antibody recognizes SBP tag. Application: Immunofluorescence Analysis: A previous lot was used by an independent laboratory in IF. (Hudson, D., et al. (2008). MBC. 19 (7):3070-3079.) Quality: Evaluated by Western Blot in pTrAP-GFP transfected Hek293 lysate. Western Blot Analysis: 0.1 ng/mL of this antibody detected SBP-tag on 10 µg of pTrAP-GFP transfected Hek293 lysate.
